# Ligand-Directed Metalation of a Gold Pyrazolate Cluster

**DOI:** 10.1021/acs.inorgchem.3c01667

**Published:** 2023-06-07

**Authors:** Ryan A. Smith, Rafal Kulmaczewski, Malcolm A. Halcrow

**Affiliations:** School of Chemistry, University of Leeds, Woodhouse Lane, Leeds LS2 9JT, United Kingdom

## Abstract

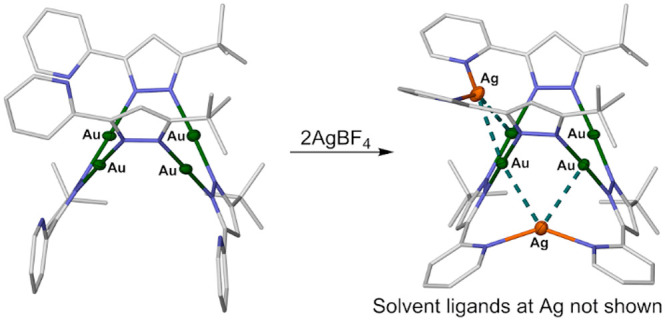

Solid “[Au*L*]” (H*L* = 3-[pyrid-2-yl]-5-*tert*butyl-1*H*-pyrazole) can be crystallized as cyclic [Au_3_(μ-*L*)_3_] and [Au_4_(μ-*L*)_4_] clusters from different solvents. The crystalline tetramer contains a square Au_4_ core with an HT:TH:TH:HT arrangement of ligand substituents, which preorganizes the cluster to chelate to additional metal ions via its pendant pyridyl groups. The addition of 0.5 equiv of AgBF_4_ to [Au*L*] yields [Ag_2_Au_4_(μ_3_-*L*)_4_][BF_4_]_2_, where two edges of the Au_4_ square are spanned by Ag^+^ ions via metallophilic Ag···Au contacts. Treatment of [Au*L*] with [Cu(NCMe)_4_]PF_6_ affords the metalloligand helicate [Cu_2_Au_2_(μ-*L*)_4_][PF_6_]_2_, via oxidation of the copper and partial fragmentation of the cluster.

## Abstract 2

Metalation of [Au_*n*_(μ-*L*)_*n*_] (H*L* = 3-[pyrid-2-yl]-5-*tert*butyl-1*H*-pyrazole; *n* = 3 or 4) with AgBF_4_ yields [Ag_2_Au_4_(μ_3_-*L*)_4_][BF_4_]_2_, where two edges of the Au_4_ square are spanned by Ag^+^ ions coordinated to its pendant pyridyl groups. Treatment of [Au_*n*_(μ-*L*)_*n*_] with [Cu(NCMe)_4_]PF_6_ affords a metalloligand helicate [Cu_2_Au_2_(μ-*L*)_4_][PF_6_]_2_, via oxidation of the copper and partial fragmentation of the cluster.

Coinage metal pyrazolate salts adopt oligomeric structures, with trimeric and tetrameric molecular and 1D polymeric structure types being well-known in the solid state.^1−4^ The [M_3_(μ-pz)_3_] (M = Cu, Ag, or Au; Hpz = 1*H*-pyrazole, or a substituted derivative) cyclic trimer is the most common motif in these compounds.^4^ These are essentially planar, notwithstanding any peripheral substituents, and often aggregate in the crystal through short M···M contacts. Such compounds can show an intense, temperature-dependent emission in the solid state,^5−10^ from transitions within the intermolecular metallophilic orbitals.^11−13^ Similarly, hybrid or soft materials based on [M_3_(μ-pz)_3_] centers can show switchable emission mediated by reversible supramolecular aggregation processes.^14−18^ Triangular [M_3_(μ-pz)_3_] centers with appropriate substituents can be π-acid hosts for aromatic guest species,^1,4,19,20^ and *D*_3*h*_-symmetric synthons in crystalline frameworks^21,22^ and in 2D coordination nanosheets.^23^

Sterically hindered 3-(pyrid-2-yl)-5-*tert*butyl-1*H*-pyrazole (H*L*) supports a number of novel metal–organic molecular architectures.^24−28^ For example, the silver chemistry of H*L* afforded a rare example of metallophilic isomerism in two polymorphs of [Ag_3_(μ-Br)(μ-*L*)_2_], and the largest known homoleptic coinage metal pyrazolate cluster [Ag_10_(μ-*L*)_8_]^2+^.^28^ We were therefore intrigued to study complexes of H*L* with other coinage metals. We report here the isolation of two clusters [Au_*n*_(μ-*L*)_*n*_] (*n* = 3 or 4) and their further reaction with other metal sources to form mixed-metal compounds. This has resulted in a rare postsynthetic metalation of a preformed gold(I) cluster with Ag(I), without inducing any further structural rearrangement.^29−36^

Dropwise addition of NBu_4_OH solution to an equimolar suspension of [AuCl(tht)] (tht = tetrahydrothiophene) and H*L*^37^ in methanol affords a clear, pale yellow solution. Storage of the filtered solution at 255 K for 3 days yields an off-white microcrystalline precipitate analyzed as [Au*L*] (**1**). The synthesis is unpredictable and sometimes yields colloidal gold rather than the desired complex **1**. Analogous reactions using different solvents and bases also suffered from this problem while giving lower yields of **1** when they worked as desired. Since most gold pyrazolates precipitate cleanly when synthesized from polar solvents, the sensitivity of this reaction might reflect the chelating N-donors in [*L*]^−^, which could be incompatible with the preferred linear coordination of gold(I) in the reaction mixture. Be that as it may, once isolated, **1** is stable under ambient conditions and soluble in weakly polar solvents.

Recrystallization of **1** from chlorinated solvents affords mixtures of colorless crystals and an amorphous material. Crystals of [Au_3_(μ-*L*)_3_] (**1a**) and [Au_4_(μ-*L*)_4_]·*x*Et_2_O (**1b**·*x*Et_2_O; *x* ≈ 0.63) were obtained from two such crystallization mixtures. The cyclic trimer molecule **1a** has crystallographic 6̅ symmetry with a planar, equilateral Au_3_ core and symmetry-imposed pyridyl group disorder (Figure 1). The three [*L*]^−^ ligands are oriented in a HT:HT:HT (H = head {pyridyl}; T = tail {*tert*butyl}) arrangement. The cyclic tetramer in **1b** lies on a general crystallographic position and has an approximately planar Au_4_ core with [*L*]^−^ ligands alternating above and below the plane of metal atoms. Interestingly, the disposition of ligands around this molecule is HT:TH:TH:HT. There are no close intramolecular steric contacts between the pyridyl or *tert*-butyl substituents that might influence the ligand arrangement in **1b**·*x*Et_2_O. However, the same isomer is a major component in solutions of **1**, as described below. In contrast, another [Au_4_(μ-pz)_4_] complex with an unsymmetric pattern of bulky pyrazole substituents adopts the more symmetrical HT:HT:HT:HT isomer in the solid state.^38^

**Figure 1:**
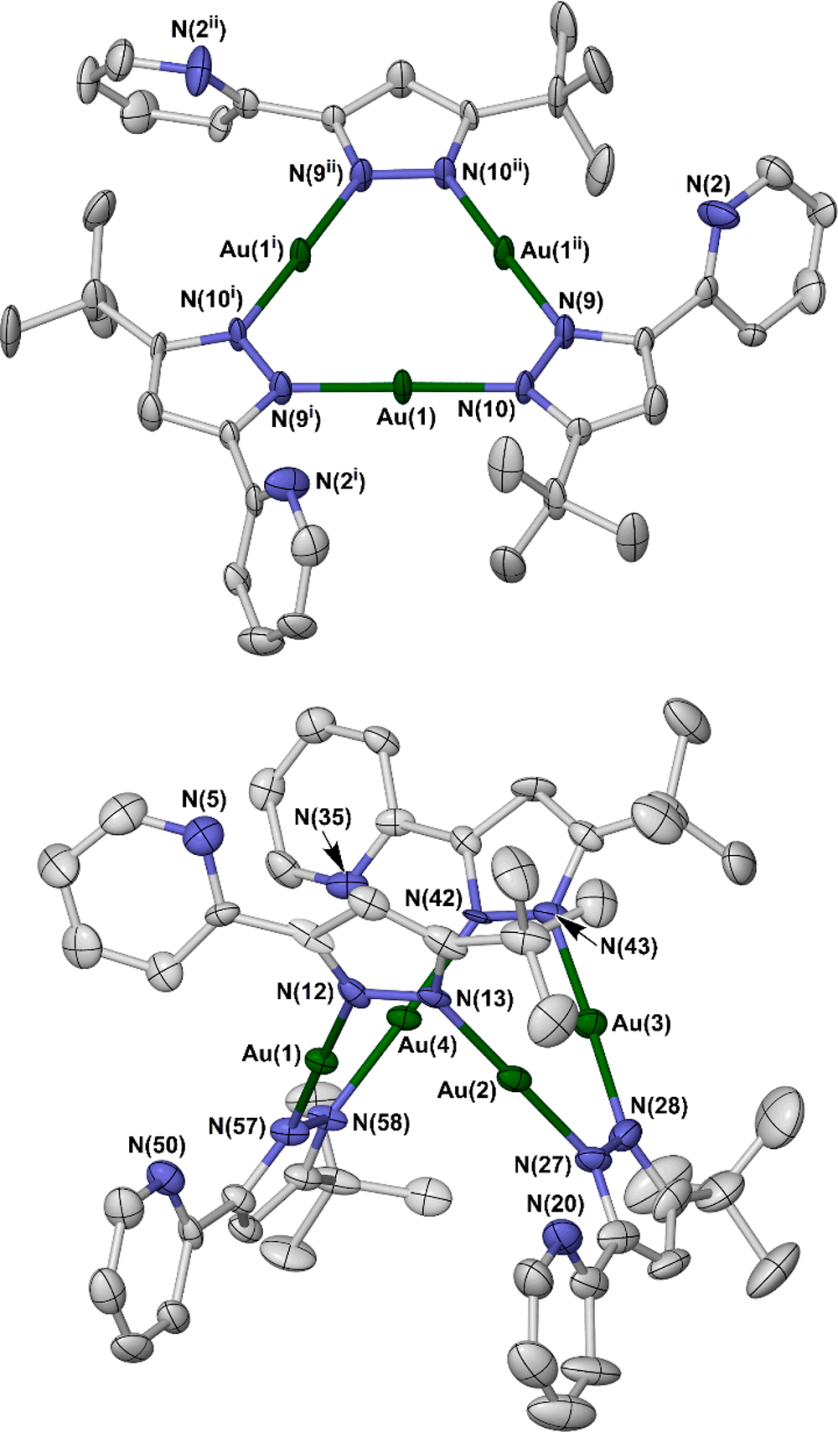
[Au_3_(μ-*L*)_3_] molecule in **1a** (top) and the [Au_4_(μ-*L*)_4_] molecule in **1b**·*x*Et_2_O (bottom). Only one of the symmetry-imposed disorder sites for the pyridyl groups in compound **1a** is shown. Displacement ellipsoids are at the 50% probability level, and H atoms are omitted for clarity. Color code: C, white; Au, green; N, blue. Symmetry codes: (i) 1 – *y*, *x* – *y*, *z*; (ii) 1 – *x* + *y*, 1 – *x*, *z*.

The Au···Au distances in **1a** are all 3.3529(7) Å, while in **1b** they range between 3.1661(8) and 3.3053(8) Å. These are typical dimensions for these classes of compounds and imply only weak interactions between the metal ions in each molecule. There are no close intermolecular Au···Au contacts in the lattices of **1a** and **1b**·*x*Et_2_O, which presumably reflects the steric bulk of their *tert*-butyl groups (Figures S3 and S4).

Bulk samples of **1** are a mixture of at least two phases determined by powder diffraction, including **1a** and a phase related to **1b**·*x*Et_2_O. Unfortunately, we have been unable to purify bulk samples of **1a** and **1b** for separate characterization.

Some other coinage metal pyrazolate complexes have also been crystallized in more than one aggregation state,^39−43^ which can exist in concentration-dependent equilibria in solution.^43−45^ However, the ESMS spectrum of **1** shows a strong molecular ion for [**1b** + H]^+^ (*m*/*z* = 1589.3473) but no peak assignable to **1a**. Hence, **1a** should be a minor component in solutions of **1**, even though it can be crystallized under some conditions. Other coinage metal pyrazolates with *tert*-butyl ligand substituents also prefer tetranuclear over trinuclear structures, probably on steric grounds.^38,46,47^ A ^1^H NMR spectrum of **1** in CDCl_3_ demonstrated the presence of three main species with one, two, and four unique *L* environments (Figures S7 and S8).^48^ For consistency with the mass spectrum, we assign these to three isomers of tetranuclear [Au_4_(μ-*L*)_4_], respectively with HT:HT:HT:HT, HT:TH:TH:HT (*i.e.*, **1b**), and HT:TH:HT:HT pyrazole substituent patterns (Chart S2).

The disposition of the ligands in **1b** places its pyridyl substituents adjacent to each other across two edges of the Au_4_ square (Figure 1). That could preorganize them to chelate to additional metal ions.^29,32,35,49−54^ We therefore explored reactions of preformed **1** with additional equivalents of other coinage metal precursors. No reaction was observed between **1** and [Au(tht)_2_]PF_6_, which gave unchanged **1** as the only isolable product. However, treatment of **1** with 0.5 equiv AgBF_4_ per the “[Au*L*]” formula unit in thf affords a new off-white product, [Ag_2_Au_4_(μ_3_-*L*)_4_][BF_4_]_2_ (**2**).^39^ Compound **2** is soluble in MeCN and MeNO_2_, but it does not form single crystals from those solvents. However, single crystals of formula **2**·*y*C_2_H_4_Cl_2_, *y* ≈ 3.6) were grown by slow evaporation of a solution of **2** in 1,2-dichloroethane, in which it is only sparingly soluble.

The structure of **2** contains a square [Au_4_(μ-*L*)_4_] moiety, whose geometry and metric parameters are almost identical to those of **1b** within experimental error. However, two adjacent edges of the square assembly are now spanned by silver ions (Figure 2). Each Ag(I) ion is coordinated by the pyridyl N-donor atom from two [*L*]^−^ ligands, as predicted, with an additional long contact to a partially occupied, disordered solvent molecule. The N–Ag–N angle is significantly bent at 135.8(4)°, which orients each silver atom toward the midpoint of a Au···Au vector. The Ag···Au distances of 2.9344(12)–2.9346(10) Å lie within the midrange for metallophilic (d^10^–d^10^) bonding interactions between those two metals.^55^ The Au···Au distances span a narrower range than in **1b**, at 3.1645(6)–3.1878(7) Å (Figure S10). The average Au···Au distance in **2** [3.1762(9) Å] is 0.0753(19) Å shorter than that in **1b** [3.2515(17) Å], implying that metalation of the Au_4_ cluster in **2** strengthens its metallophilic bonding to a small degree. The Au–N distances in **2** are equal to each other and to those in **1b**, within experimental error. Hence, metalation of **2** has no detectable influence on the Au–[pyrazolate] bonding.

**Figure 2:**
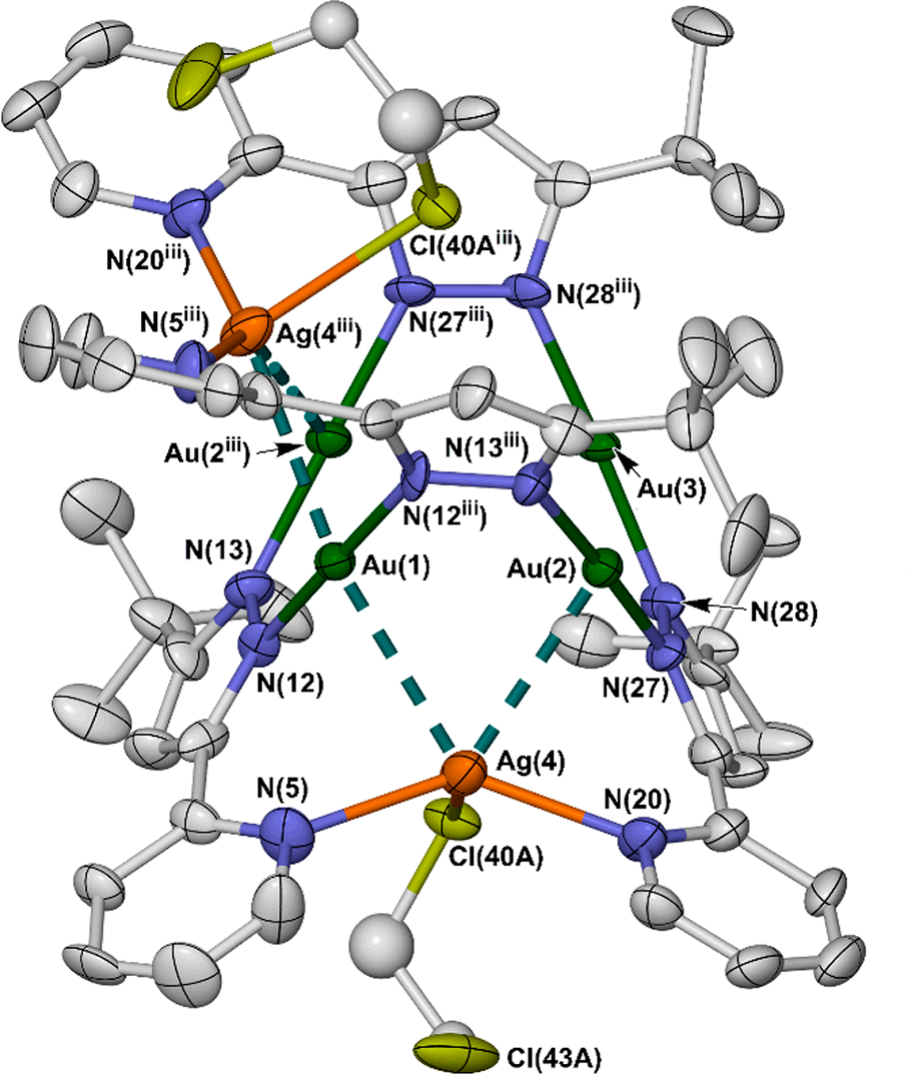
[Ag_2_Au_4_(μ_3_-*L*)_4_(ClC_2_H_4_Cl)_1.2_]^2+^ dication in **2**·*y*C_2_H_4_Cl_2_. The short Ag···Au contacts are plotted as dotted lines, and only the major disorder site for the part-occupied 1,2-dichloroethane ligand is shown. Other details are as in Figure 1. Color code: C, white; Ag, orange; Au, green; Cl, yellow; N, blue. Symmetry code: (iii).

A ^1^H NMR spectrum of **2** in CD_3_CN showed a single species with two equally populated *L* environments, which is the symmetry expected for both **1b** and **2** (Figure S13). Silver complexes of heterocyclic ligands are often labile in solution.^28,43−45,56−60^ However, the pyridyl *H*^3^ resonances in **2** lie 0.5–0.6 ppm upfield compared to **1b**, implying that silver coordination to those residues is retained in the sample. Consistent with that, the ESMS spectrum of **2** contains a strong peak assigned to [AgAu_4_*L*_4_]^+^ (*m*/*z* = 1697.2449), as well as a peak from demetalated **1b** (Figure S14).

Reaction of **1** with 0.5 equiv of [Cu(NCMe)_4_]PF_6_ under similar conditions used to synthesize **2** afforded strongly colored solutions implying oxidation of the copper reagent to copper(II). Solid products from these reactions were sparingly soluble, but milligram quantities of red-green dichroic crystals of [Cu_2_Au_2_(μ-*L*)_4_][PF_6_]_2_·*z*Et_2_O (**3**·*z*Et_2_O; *z* ≈ 0.8) were crystallized from 2,2,2-trifluoroethanol/diethyl ether.

The complex in **3** contains a butterfly Cu_2_Au_2_ core with Cu atoms at the wingtips linked to a Au_2_ hinge fragment (Figure 3). The copper ions are four-coordinate to two chelating [*L*]^−^ ligands, with a substantial tetrahedral distortion (τ_4_ = 0.43).^61^ The ligands bridge through their pyrazolate groups to near-linear gold ions. These form a Au···Au distance of 2.9483(4) Å, implying a significant aurophilic interaction^55,62^ but are well isolated from the copper atoms [Au(1)···Cu(2) = 3.8897(7) Å]. The disposition of the [Au*L*_2_]^−^ metalloligands linking the copper ions affords an unusual type of [2 + 2] helicate conformation;^63,64^ the centrosymmetric crystal lattice contains an equal ratio of Δ and Λ helical molecules.

**Figure 3:**
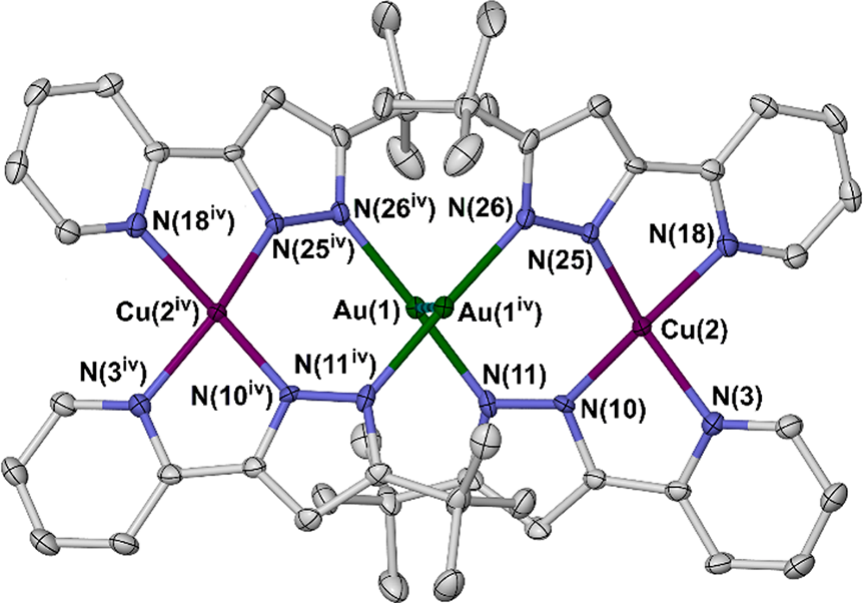
[Cu_2_Au_2_(μ-*L*)_4_]^2+^ dication in **3**·*z*Et_2_O. The short Au···Au contact is plotted as a dashed line. Other details as for Figure 1. Color code: C, white; Au, green; Cu, purple; and N, blue. Symmetry code: (iv) 1/2 – *x*, *y*, 1/2 – *z*.

The absorption spectra of **1** and **2** in MeCN at 298 K are similar to H*L*,^37^ being featureless in the visible region but with an envelope of intense pyridyl π–π* transitions around λ_max_ = 258 nm. Excitation of **1** at 270 nm yields an intense structured emission with maxima at λ_max_ = 337 and 385 nm, which is probably ligand-centered,^37,65^ and a weaker emission at λ_max_ = 654 nm (Figures 4 and S18). This resembles the orange emission shown by solutions of [Au_4_(pz^*t*Bu2^)_4_] (Hpz^*t*Bu2^ = 3,5-di(*tert*butyl)-1*H*-pyrazole),^46^ which arises from metal-to-metal charge transfer (MMCT) transitions within the metallophilic Au···Au orbital manifold.^12,13,33,47^ The emission spectrum of **2** (λ_ex_ = 250 nm) resembles that of **1**, but the main visible emission is slightly red-shifted at λ_max_ = 667 nm. The spectrum also has weak additional features in the visible region which may be vibrational structure on the emission bands, reflecting the greater conformational rigidity of **2**.^35^

**Figure 4:**
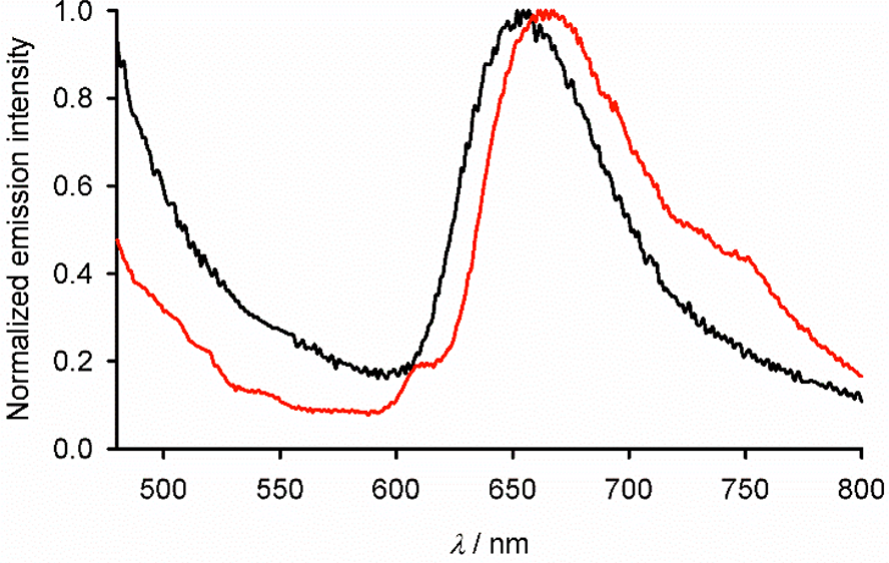
Normalized visible emission spectra of **1** (black; λ_ex_ = 270 nm) and **2** (red; λ_ex_ = 250 nm) in a MeCN solution at 298 K.

A number of [{Au_3_(μ-L)_3_}_2_Ag]^+^ clusters have been reported, where [L]^−^ is a substituted pyrazolate or a *C*,*N*-donor 1,2-bridging ligand. These contain a silver(I) ion sandwiched by two cyclic trigold(I) metalloligands, through unsupported Au···Ag metallophilic interactions.^30−34^ The sandwich assemblies can show enhanced room-temperature phosphorescence compared to the trigold precursors,^33,34^ which has been exploited in emissive soft materials^14−16^ and silver ion sensors^17,18^ containing embedded trigold clusters. Higher nuclearity [Au_*n*_(μ-L)_*n*_] (*n* > 3) complexes are less common, but our synthesis of **2** shows that they can also be decorated with silver ions, using pendant ligand substituents to direct the metalation.^35,36^ This is a promising strategy toward heterometallic pyrazolate clusters of gold and other coinage metals.^32,35^

## Data Availability

Experimental data sets associated with this paper are available from the University of Leeds library (10.5518/1361).

## Supporting Information Available

Synthetic details and characterization data for **1**–**3**; experimental data, refinement procedures, and tabulated metric parameters for the crystal structure determinations, additional crystallographic figures, and other solid state and solution characterization data (PDF)
