# Supplementary material for: Ligand-Directed Metalation of a Gold Pyrazolate Cluster
Source: Inorg Chem. 2023 Jun 7;62(24):9300–5. doi: 10.1021/acs.inorgchem.3c01667 (PMC10283019; doi:10.1021/acs.inorgchem.3c01667)
Supplement: Supplementary file 1 — ic3c01667_si_001.pdf [file ic3c01667_si_001.pdf]

Supplementary Information

Ligand-Directed Metalation of a Gold Pyrazolate Cluster

Ryan A. Smith, Rafal Kulmaczewski and Malcolm A. Halcrow*

School of Chemistry, University of Leeds, Woodhouse Lane, Leeds LS2 9JT, UK.
E-mail: m.a.halcrow@leeds.ac.uk

		Page
**Chart S1**	The complexes referred to in this study.	S2
**Experimental Section**		S2
**Table S1**	Experimental data for the single crystal structure determinations in this work.	S5
**Figure S1**	View of the formula unit in **1a**, showing the full atom numbering scheme.	S6
**Figure S2**	View of the formula unit in **1b**·$x$$Et_2O$, showing the full atom numbering scheme.	S7
**Table S2**	Selected interatomic distances and angles in **1a** and **1b**·$x$$Et_2O$.	S8
**Figure S3**	Packing diagram of **1a**, showing its Au_3 cluster cores are well-separated in the lattice.	S8
**Figure S4**	Alternative packing diagram of **1a**.	S9
**Figure S5**	X-ray powder diffraction pattern of **1**.	S10
**Figure S6**	Electrospray mass spectrum of **1**.	S10
**Figure S7**	^{1}H NMR spectrum of **1**.	S11
**Figure S8**	Expansions of the ^{1}H NMR spectrum of **1**.	S12
**Chart S2**	The species proposed to be the three main components in the NMR spectrum of **1**.	S13
**Figure S9**	View of the formula unit in **2**·$y$$C_2H_4Cl_2$.	S14
**Table S3**	Selected interatomic distances and angles in **2**·$y$$C_2H_4Cl_2$.	S14
**Figure S10**	Comparison of the Au_4 core in **1b**·$x$$Et_2O$, and the Ag_2Au_4 core in **2**·$y$$C_2H_4Cl_2$.	S15
**Figure S11**	Packing diagram of **2**·$y$$C_2H_4Cl_2$.	S16
**Figure S12**	Space-filling packing diagram of **2**·$y$$C_2H_4Cl_2$.	S17
**Figure S13**	^{1}H NMR spectrum of **2**.	S18
**Figure S14**	Electrospray mass spectrum of **2**.	S19
**Figure S15**	View of the formula unit in **3**·$z$$Et_2O$.	S20
**Table S4**	Selected interatomic distances and angles in **3**·$z$$Et_2O$.	S21
**Figure S16**	View of the $[Cu_2Au_2(\mu\text{-}L)_4]^{2+}$ molecule in **3**·$z$$Et_2O$, showing its helicate conformation.	S21
**Figure S17**	Packing diagram of **3**·$z$$Et_2O$, showing the solvent-filled channels in the lattice.	S22
**Figure S18**	Normalized absorption and emission spectra for **1** and **2** in MeCN solution.	S23
**References**		S24

Chart S1 The complexes referred to in this study.

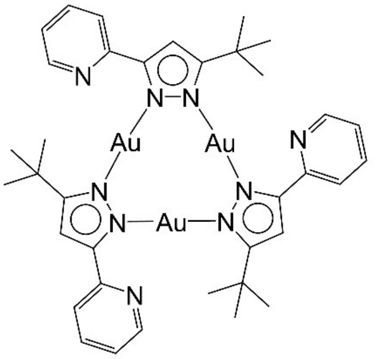

1a

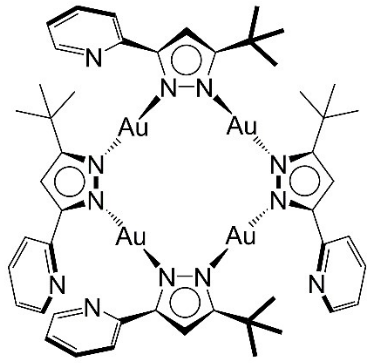

1b

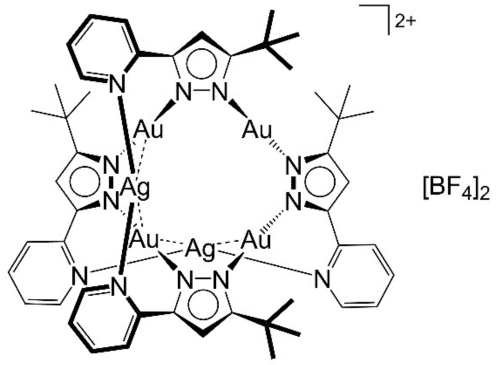

2

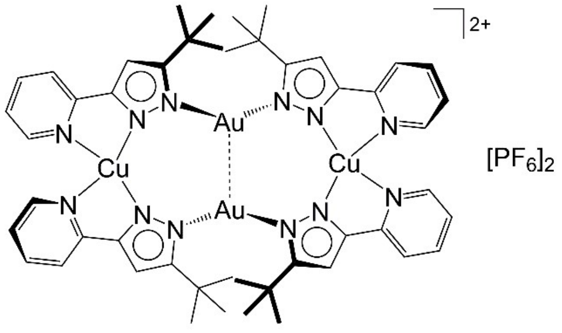

3

Synthetic Procedures and Characterization Data for the Compounds in this Work.

The ligand H*L* [1] and the coinage metal starting materials [AuCl(tht)] (tht = tetrahydrothiophene),[2] $[Au(tht)_2]PF_6$[3] and $[Cu(NCMe)_4]PF_6$[4] were prepared by the literature procedures. All other reagents were purchased commercially and used as supplied. Reactions were performed under ambient conditions using undried AR-grade solvents.

Synthesis of $[Au_n(L)_n]$ (1; *n* = 3, 1a; *n* = 4, 1b). [AuCl(tht)] (0.35 g, 1.0 mmol) and H*L* (0.20 g, 1.0 mmol) were suspended in MeOH (50 cm^3). Dropwise addition of ^{n}BuNOH (1.0 M solution in methanol; 1.0 cm^3, 1.0 mmol) to the stirred mixture resulted in rapid dissolution of the solid, yielding a pale yellow solution with a small amount of dark precipitate. The solid was removed by filtration, and the filtrate was stored at 255 K for 3 days. This afforded **1** as an off-white microcrystalline solid, which was collected and washed with methanol and diethyl ether. The product was analyzed without further purification. Yield: 0.33 g, 83 %. Elem anal. Found (calcd) for $[C_{12}H_{14}AuN_3]_n$ C 36.3 (36.3), H 3.50 (3.55), N 10.5 (10.6) %. ES-MS *m*/*z* 1589.3473 (100, calcd for $[Au_4(L)_4+H]^+$ 1589.3491). ^{1}H NMR ($CDCl_3$): species 1 (**1a**, one *L* environment) δ 1.37 (s, 27H, C{CH_3}$_3$), 6.79 (s, 3H, Pz H^4), 7.29 (ddd, 3H, Py H^5), 7.73 (pseudo-dt, 3H, Py H^4), 7.96 (ddd, 3H, Py H^3), 8.71 (ddd, 3H, Py H^6); species 2 (**1b**, two equally populated *L* environments) δ 1.58 (s, 36 H, 2x C{CH_3}$_3$), 6.89 and 6.92 (both s, 2H, Pz H^4), 7.14 (m, 4H, 2x Py H^5), 7.53 and 7.57 (both pseudo-dt, 2H, Py H^4), 8.25 and 8.41 (both d, 2H, Py H^3), 8.56 (br d, 4H, 2x Py H^6). A third species with four equally populated *L* environments is also visible from its *tert*butyl and Pz H^4 singlet resonances, but its other peaks are obscured by the other two, more intense species (Figure S8).

On some occasions, this procedure simply afforded colloidal gold when the mixture was left to stand at 255 K. Colloidal gold also formed if the solution was concentrated on a rotary evaporator before the crystallization step. Using THF as the reaction solvent or triethylamine or NaH as the base, which have been employed successfully in other gold pyrazolate complex syntheses,[5] did not give more reliable results.

Synthesis of [Au_4Ag_2(*L*)$_4$][BF_4]$_2$ (2). A mixture of **1** (0.095 g, 0.24 mmol) and $AgBF_4$ (0.026 g, 0.12 mmol) was dissolved in THF (50 cm^3). After stirring for 30 mins, the solution was stored at 255 K yielding an off-white precipitate, which was collected and dried *in vacuo*. Yield: 0.092 g, 20 %. %. Elem anal. Found (calcd) for $C_{48}H_{56}Ag_2Au_4B_2F_8N_{12}$ C 28.9 (29.1), H 2.90 (2.85), N 8.30 (8.50) %. ES-MS *m/z* 1589.3465 (100, calcd for [Au_4(*L*)$_4$+H]$^+$ 1589.3491), 1697.2449 (20, calcd for [Au_4Ag(*L*)$_4$]$^+$ 1697.2465). ^{1}H NMR (CD_3CN): δ 1.43 and 1.60 (both s, 18H, C{CH_3}$_3$), 6.99 and 7.03 (both s, 2H, Pz H^4), 7.50 and 7.56 (both ddd, 2H, Py H^5), 7.77 and 7.84 (both d, 2H, Py H^3), 7.98 and 8.05 (pseudo-dt, 2H, Py H^4), 8.70 and 8.78 (both br d, 2H, Py H^6).

While **2** is moderately soluble in $MeNO_2$, MeCN and acetone, it does not form single crystals from those solvents. Small quantities of single crystals were obtained, however, by slow evaporation of a solution of the complex in 1,2-dichloroethane, in which it is only sparingly soluble. Elem anal. Found (calcd) for $C_{48}H_{56}Ag_2Au_4B_2F_8N_{12}\cdot 4C_2H_4Cl_2$ C 28.0 (28.3), H 3.10 (3.06), N 6.90 (7.08) %. The presence of 1,2-dichloroethane in the porous crystals was confirmed crystallographically.

Synthesis of [Cu_2Au_2(*L*)$_4$][PF_6]$_2$ (3). Solid **1** (0.040 g, 0.1 mmol) and [Cu(NCMe)$_4$]PF_6 (0.019 g, 0.05 mmol) were dissolved in THF (20 cm^3). The solution was stirred for 1 hr, turning from colorless to dark purple. The solvent was then removed and the resultant black oil was redissolved in 1,2-dichloroethane. Slow evaporation of the filtered solution at room temperature yielded an amorphous purple precipitate, which was collected and dried. Yield: 0.091 g, 69 %. Milligram amounts could be crystallized from 2,2,2-trifluoroethanol/diethyl ether, giving rhombic red/green dichroic crystals which decompose through solvent loss on exposure to air. Elem anal. Found (calcd) for $C_{48}H_{56}Au_2Cu_2F_{12}N_{12}P_2\cdot 2H_2O$ C 34.9 (35.0), H 3.30 (3.67), N, 10.0 (10.2) %.

Experimental Procedures for the Crystal Structure Determinations in this Work

Colorless rod-shaped crystals of [Au_3(μ-*L*)$_3$] (**1a**) were grown by slow evaporation of a solution of **1** in a dichloromethane/tetrahydrofuran solvent mixture, while slow diffusion of diethyl ether vapor into a solution of **1** in 1:1 1,2-dichloroethane:hexane afforded of **1b**·*x*Et_2O as colorless rhombic prisms. Colorless crystals of **2**·*y*$C_2H_4Cl_2$ were grown by slow evaporation of a solution of **2** in 1,2-dichloroethane. Diffusion of diethyl ether vapor into a 2,2,2-trifluoroethanol solution of **3** yielded red-green dichroic prisms of **3**·*z*Et_2O.

Diffraction data were collected with an Agilent Supernova diffractometer using monochromated Mo-K_α radiation (λ = 0.71073 Å). The diffractometer was fitted with an Oxford Cryostream low temperature device. The structures were solved by direct methods (*SHELX-TL*[6]), and developed by cycles of full least-squares refinement on F^2 and difference Fourier syntheses (*SHELXL2018*[7]). Crystallographic Figures were produced using *XSEED*,[8] which incorporates *POVRAY*,[9] while publication materials were prepared using *OLEX2*.[10]

Experimental data for the crystal structures are listed in Table S1. Unless otherwise stated, all fully occupied non-H atoms in the structures were refined anisotropically, and H atoms were placed in calculated positions and refined using a riding model.

Crystallographic refinement of [Au_3(μ-*L*)$_3$] (1a). The asymmetric unit contains one-third of the trinuclear complex spanning the crystallographic $\bar{6}$ axis at ⅔, ⅓, ¾. Atoms Au(1), C(2) and C(8)-C(14) all lie on the mirror plane *x*, *y*, ¾. The unique *tert*butyl substituent is crystallographically ordered across this mirror plane, but the pyridyl group does not lie in the mirror plane and is thus disordered about it. N–C and C–C distance restraints were required for the pyridyl group to produce a reasonable geometry. All non-H atoms, including the disordered pyridyl group, were refined anisotropically. The highest residual Fourier peak of 1.9 *e*.Å^{-3} lies at the center of the pyrazolyl ring.

Crystallographic refinement of [Au_4(*μ-L*)$_4$]·*x*Et_2O (1b·*x*Et_2O; *x* ≈ 0.625). In addition to the molecule of interest, two half-diethyl ether molecules with crystallographically-imposed C_2 symmetry were also located. These solvent sites were allocated estimated occupancies of 0.75 and 0.5, based on their displacement parameters, and refined without restraints. Slightly high displacement parameters on some peripheral C atoms may imply a small degree of librational disorder on those substituents (possibly associated with the presence or absence of the neighboring solvent site). Attempts to model this made no difference to the overall refinement however, so these atoms were left as ordered in the final least squares cycles. There are sixteen residual Fourier peaks of between 1.2-3.7 *e*.Å^{-3}, which are all associated with the Au_4 core of the complex.

Crystallographic refinement of [Ag_2Au_4(*μ*-L)$_4$][BF_4]$_2$·*y*$C_2H_4Cl_2$ (2·*y*$C_2H_4Cl_2$; *y* ≈ 3.6). The asymmetric unit contains half a formula unit, with Au1 and Au3 lying on the crystallographic C_2 axis 0, *y*, ¼. There is also one unique BF_4^- ion, that is disordered over two equally occupied sites, and a three-fold disordered dichloroethane molecule with a total occupancy of 0.6, that is close to the disordered anion and to Ag(1). The following fixed restraints were applied in the model: for the BF_4^- ion B–F = 1.37(2) and F...F = 2.24(2) Å; and for the dichloroethane, C–Cl = 1.77(2), C–C = 1.51(2) and 1,3-C...Cl = 2.68(2) Å. The crystal lattice contains channels running parallel to *c*, that are filled with disordered solvent. A *SQUEEZE* analysis detected 949 Å^{-3} void space per unit cell, 13 % of the unit cell volume, which was filled with 488 electrons-worth of unresolved electron density.[11] That corresponds to *ca.* 2.4 equiv of additional 1,2-dichloroethane per formula unit (50 electrons per solvent molecule). That formula is used for the density and *F*(000) calculations in the cif file.

The *SQUEEZE*d dataset was used in the final least squares cycles. All fully occupied non-H atoms, plus two 0.3-occupied partial Cl atoms from the resolved dichloroethane solvent site, were refined anisotropically. The ten largest residual Fourier peaks of 2.0-4.3 *e* Å^{-3} are associated with Au atoms in the model, while other peaks between 1.5-1.9 *e* Å^{-3} either lie within the disordered solvent ligand bound to Ag(4), or close to Au atoms as before.

Crystallographic refinement of [Cu_2Au_2(*μ*-L)$_4$][PF_6]$_2$·*z*Et_2O (3·*z*Et_2O; *z* ≈ 0.8). The asymmetric unit contains half a formula unit, with a crystallographic C_2 axis at the center of the Au(1)...Au(1^{iv}) vector [symmetry code (iv): ½–*x*, *y*, ½–*z*]. As well as half the complex molecule and one unique PF_6^- ion, there is a partial diethyl ether molecule near the C_2 axis ¾, *y*, ¼. This was refined with occupancy 0.4, using the following fixed distance restraints to ensure a reasonable geometry: C–C = 1.52(2), C–O = 1.43(2), 1,3-C...C = 2.34(2) and 1,3-C...O = 2.41(2) Å. There are nine residual Fourier peaks of between 1.0-3.0 *e* Å^{-3}, which are all <1.2 Å from Au(1).

CCDC 2238818-2238821 contain the supplementary crystallographic data for this paper (Table S1). These data can be obtained free of charge from The Cambridge Crystallographic Data Centre via www.ccdc.cam.ac.uk/data_request/cif.

Other measurements. Elemental microanalyses were performed by the University of Leeds School of Chemistry microanalytical service. IR spectra were run using a Nicolet Paragon 1000 spectrometer, using nujol mull samples held between NaCl windows.

All solution measurements were performed using freshly prepared solution samples. Electrospray mass spectra were obtained on a Bruker MaXis spectrometer, from MeCN feed solutions. ^{1}H NMR spectra employed a Bruker DPX300 spectrometer operating at 300.2 MHz. UV/vis spectra were measured using a PerkinElmer Lambda 900 spectrophotometer. Fluorescence measurements under ambient conditions were obtained using a Horiba Fluoromax 3 fluorimeter with constant slit widths of 2 nm. A range of excitation wavelengths were sampled, and the data quoted are for the excitation wavelength that led to the most intense emission for each compound. Sample concentrations for the fluorescence spectra were 1.0 × 10^{-5} mol dm^{-3}.

Table S1 Experimental data for the single crystal structure determinations in this work.

	$[Au_3(\mu\text{-}L)_3]$ (**1a**)	$[Au_4(\mu\text{-}L)_4]\cdot x Et_2O$ (**1b**$\cdot x$ Et_2O; $x \approx 0.63$)	$[Ag_2Au_4(\mu\text{-}L)_4][BF_4]_2\cdot y C_2H_4Cl_2$ (**2**$\cdot y C_2H_4Cl_2$; $y \approx 3.6$)	$[Cu_2Au_2(\mu\text{-}L)_4][PF_6]_2\cdot z$ Et_2O (**3**$\cdot z Et_2O$; $z \approx 0.8$)
Molecular formula	$C_{36}H_{42}Au_3N_9$	$C_{50.5}H_{62.3}Au_4N_{12}O_{0.63}$	$C_{55.2}H_{70.4}Ag_2Au_4B_2Cl_{7.2}F_8N_{12}$	$C_{51.20}H_{64}Au_2Cu_2F_{12}N_{12}O_{0.80}P_2$
M_r	1191.69	1635.24	2334.50	1671.30
Crystal class	Hexagonal	Orthorhombic	Monoclinic	Monoclinic
Space group	$P6_3/m$	$Fdd2$	$C2/c$	$P2/n$
a (Å)	15.0533(15)	14.7653(2)	26.192(2)	9.0809(4)
b (Å)	–	42.3597(6)	14.6735(8)	15.5065(7)
c (Å)	9.2391(14)	35.2243(4)	19.1979(19)	23.3510(11)
α (°)	–	–	–	–
β (°)	–	–	102.964(9)	99.553(4)
γ (°)	–	–	–	–
V (Å^3)	1813.1(4)	22031.2(5)	7190.2(10)	3242.5(3)
Z	2	16	4	2
μ (Mo-K$_\alpha$) (mm^{-1})	12.147	10.667	8.929	5.285
T (K)	120(2)	120(2)	120(2)	120(2)
Measured reflections	5727	75469	27831	22826
Independent reflections	1494	12043	7903	6988
R_{int}	0.064	0.056	0.043	0.041
R_1 [$I > 2\sigma(I)$][a]	0.044	0.042	0.064	0.041
wR_2 (all data)[b]	0.092	0.098	0.151	0.100
Goodness of fit	1.187	1.050	1.050	1.122
Flack parameter	–	–0.028(5)	–	–
CCDC	2238818	2238819	2238820	2238821

[a] $R = \Sigma[|F_o| - |F_c|] / \Sigma|F_o|$ [b] $wR = [\Sigma w(F_o^2 - F_c^2) / \Sigma wF_o^4]^{1/2}$

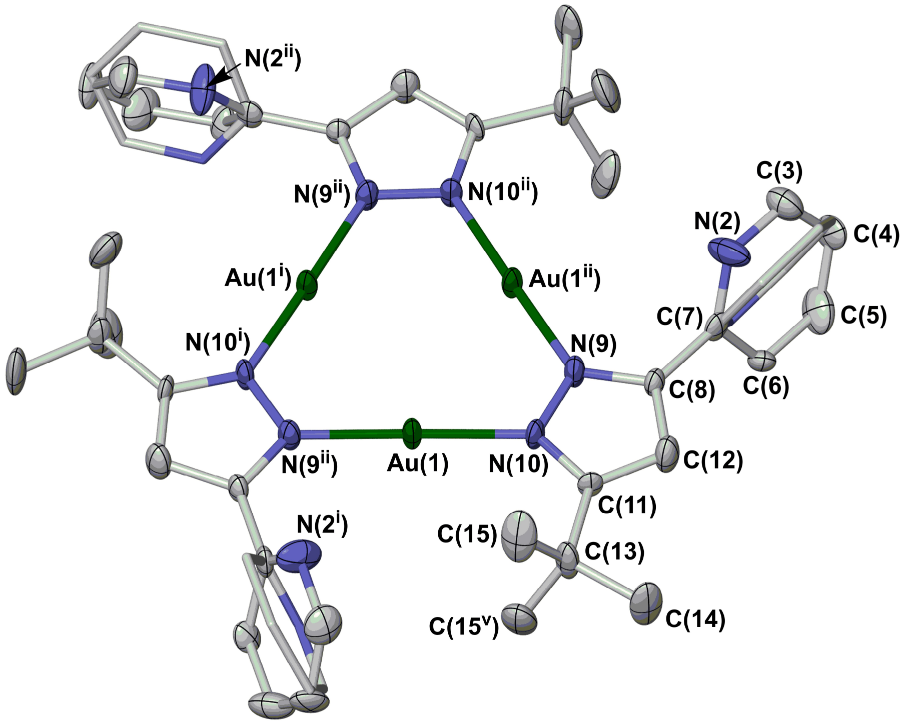

Figure S1 View of the formula unit in **1a**, showing the full atom numbering scheme. Both symmetry-related disorder orientations for the pyridyl groups are shown, with one disorder site for each pyridine ring being de-emphasized for clarity. Displacement ellipsoids are at the 50 % probability level, and atoms are omitted. Symmetry codes: (i) 1−*y*, *x*−*y*, *z*; (ii) 1−*x*+*y*, 1−*x*, *z*; (v) *x*, *y*, $^{3}/_{2}$−*z*.

Color code: C, white; Au, green; N, blue; O, red.

The molecule has crystallographic C_{3h} symmetry. The ligand *tert*-butyl groups are crystallographically ordered across the molecular mirror plane, but the pyridyl groups lie out of the mirror plane and are disordered about it.

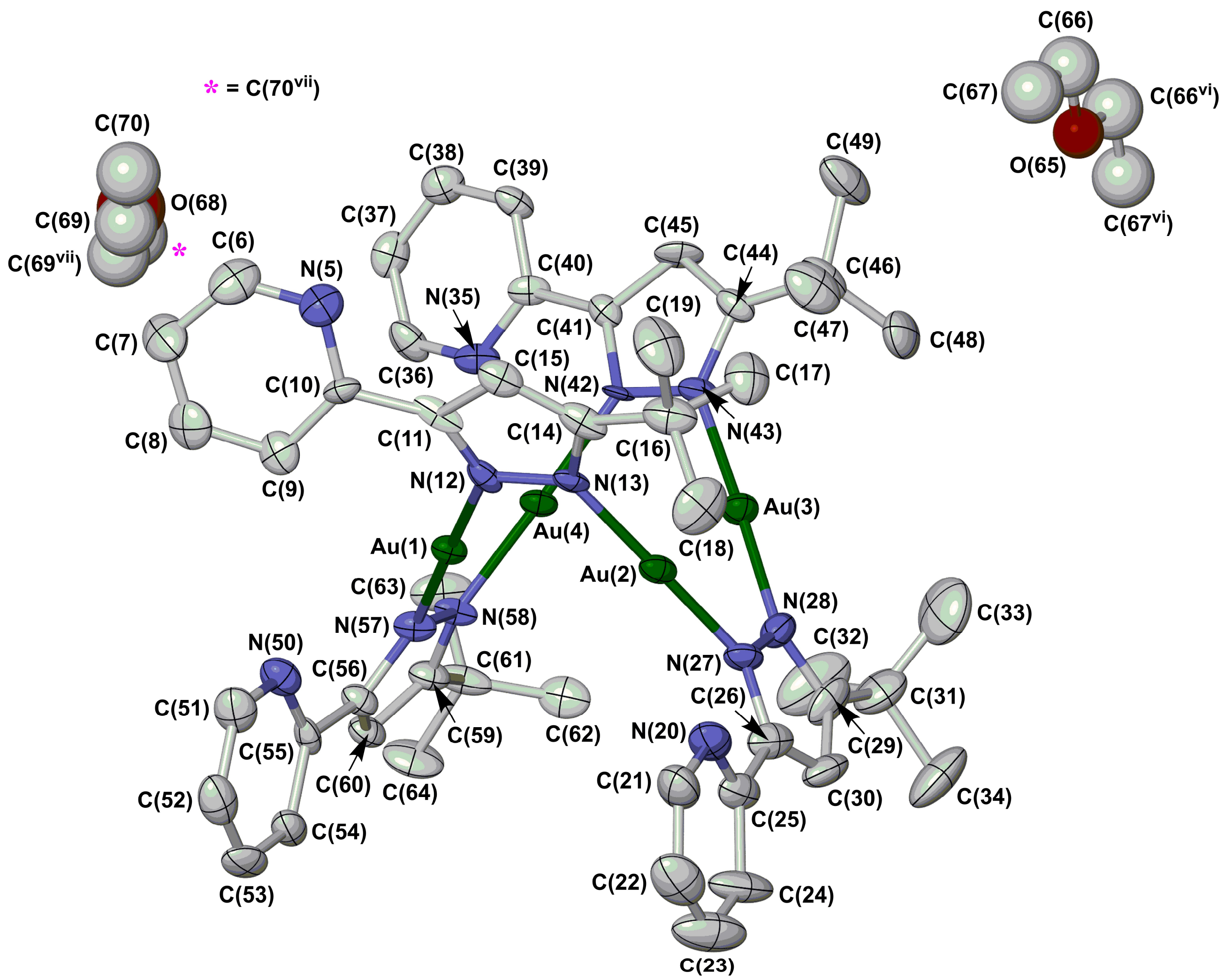

Figure S2 View of the formula unit in **1b**·xEt$_2$O, showing the full atom numbering scheme. Only one of the symmetry-related disorder sites for the pyridyl groups in **1a** is shown. Displacement ellipsoids are at the 50 % probability level, and atoms are omitted for clarity. Symmetry codes: (vi) $1-x$, $-y$, z; (vii) $^{5}/_{2}-x$, $^{1}/_{2}-y$, z.

Color code: C, white; Au, green; N, blue; O, red.

Both diethyl ether half-molecules have crystallographic C_2 symmetry, with their O atoms lying on those symmetry axes. The occupancies of molecules O(65)-C(67) and O(68)-C(70) in the model are 0.375 and 0.25, respectively.

Table S2 Selected interatomic distances and angles in **1a** and **1b**·*x*Et_2O (Å, °). See Figures S1 and S2 for the atom number schemes employed.

**1a**			
Au(1)–N(9^{ii})	2.002(8)	Au(1)···Au(1^{ii})	3.3529(7)
Au(1)–N(10)	2.010(8)		
N(9^{ii})–Au(1)–N(10)	179.1(3)		
**1b**·*x*Et_2O			
Au(1)–N(12)	2.015(13)	Au(4)–N(42)	2.007(11)
Au(1)–N(57)	2.008(12)	Au(4)–N(58)	2.015(12)
Au(2)–N(13)	2.014(12)	Au(1)···Au(2)	3.3053(8)
Au(2)–N(27)	2.016(14)	Au(2)···Au(3)	3.1661(8)
Au(3)–N(28)	1.989(14)	Au(3)···Au(4)	3.2500(9)
Au(3)–N(43)	2.000(12)	Au(4)···Au(1)	3.2847(8)
N(12)–Au(1)–N(58)	178.5(5)	N(27)–Au(3)–N(43)	177.0(5)
N(13)–Au(2)–N(28)	179.1(5)	N(42)–Au(4)–N(57)	178.5(5)

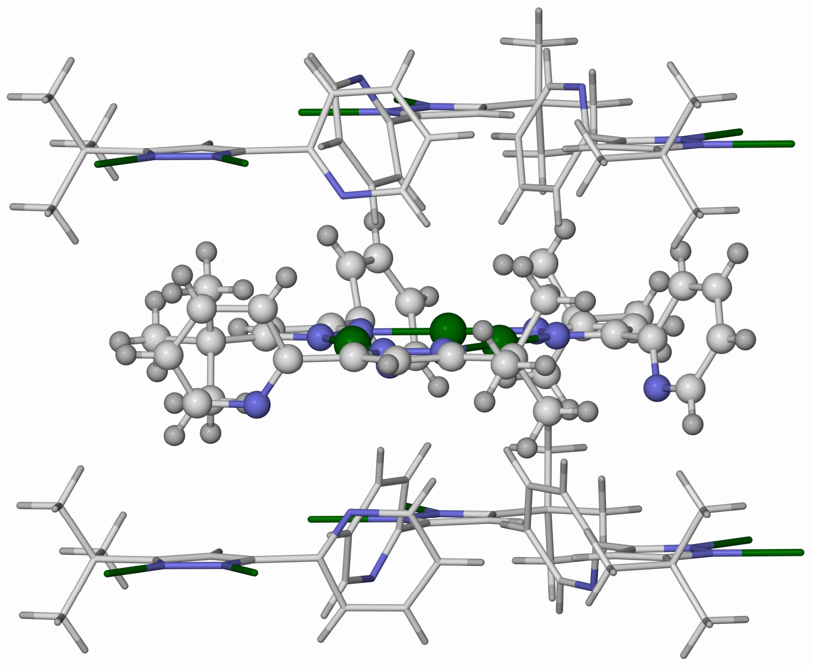

Figure S3 Partial packing diagram of **1a**, showing that the Au_3 cores of the complex molecules are overlaid by the pyridyl groups in adjacent molecular layers. Only one symmetry-related disorder orientation is shown for each pyridyl group, and all atoms have arbitrary radii.

Color code: C, white; Au, green; N, blue.

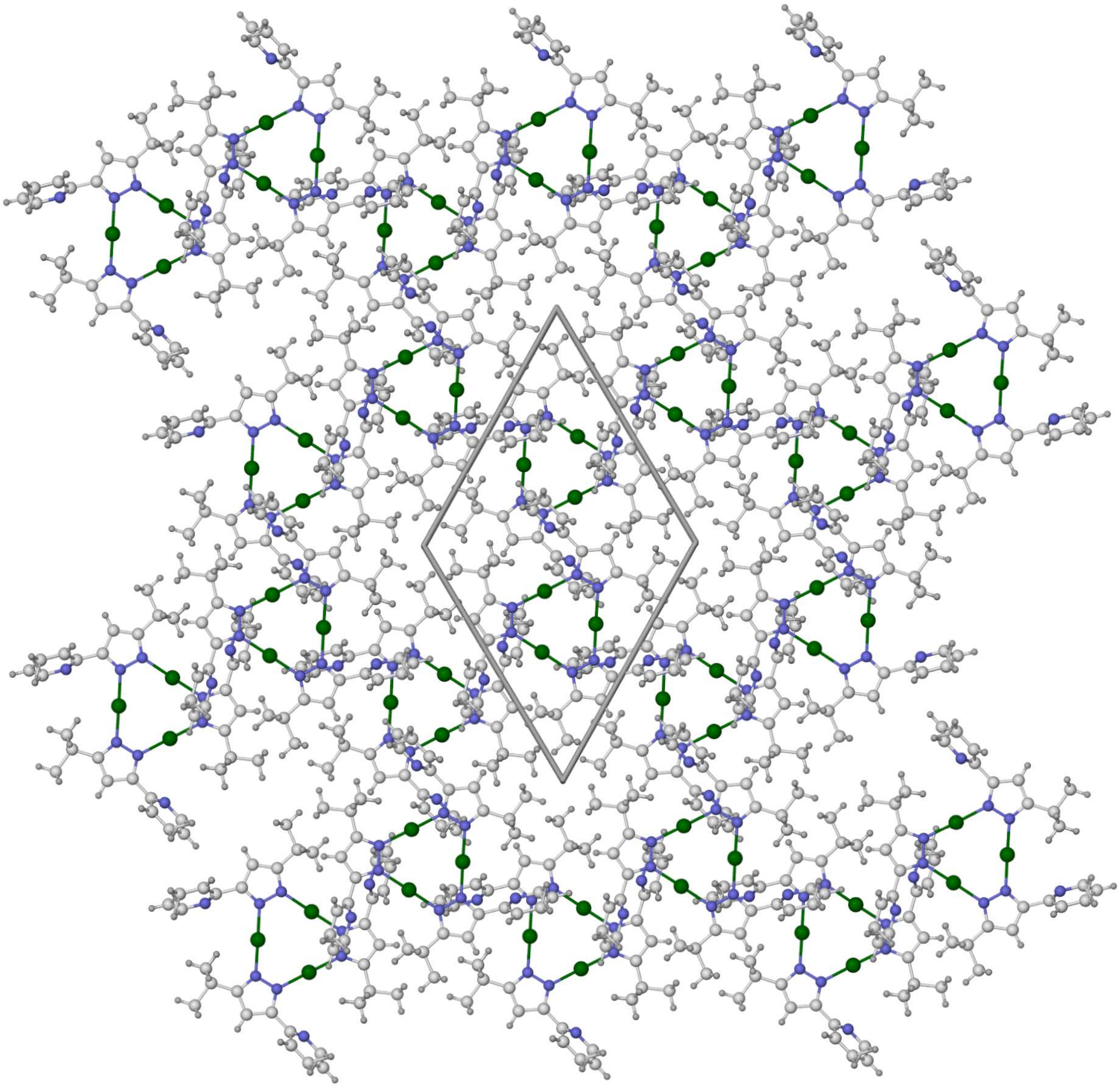

Figure S4 Packing diagram of **1a**, showing that the Au_3 cores of the complex molecules are well separated in the lattice. The view is parallel to the [001] crystal vector, and all atoms have arbitrary radii.

Color code: C, white; Au, green; N, blue.

The closest inter-trimer Au···Au distance is Au(1)···Au(1^{viii}) = 7.1973(8) Å [symmetry code (viii): $1–x$, $–y$, $^1/_2+z$].

No packing diagram for **1b**·$x$$Et_2O$ is included in this ESI since its unit cell is large (Z = 16), the packing is irregular and no informative view of the lattice could be found. However, the closest inter-tetramer Au···Au distance is Au(1)···Au(4^{ix}) = 7.5063(7) Å [symmetry code (ix): $x–^1/_4$, $^1/_4–y$, $z–^1/_4$].

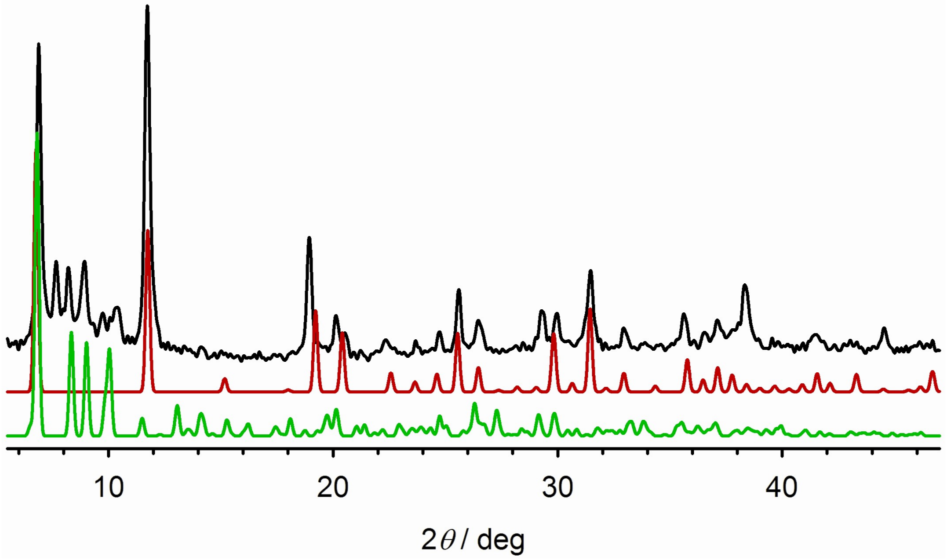

Figure S5 X-ray powder pattern of a bulk sample of **1** (black), and simulated patterns based on the crystal structures of **1a** (red) and **1b**·xEt$_2$O (green).

The bulk material clearly contains **1a**, and reflections resembling the **1b**·xEt$_2$O phase are also apparent. Peaks that do not correspond to either phase are also present at low angle, however.

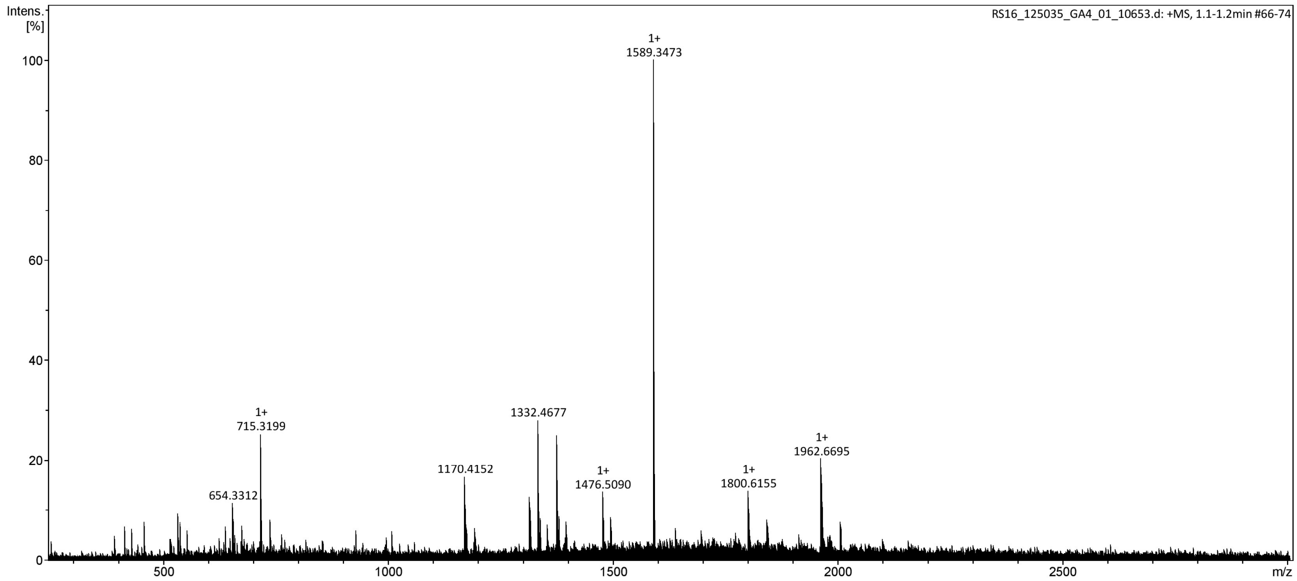

Figure S6 Electrospray mass spectrum of **1**.

The principal peak at m/z = 1589.3473 corresponds to the molecular ion for **1b**, [HAu$_4$(L)$_4$]$^+$ (calcd 1589.3491). There is significant fragmentation, which could not be assigned. However no peaks unambiguously corresponding to **1a** can be identified in the spectrum (*eg* [HAu$_3$(L)$_3$]$^+$, calcd m/z = 1192.2638; [NaAu$_3$(L)$_3$]$^+$, calcd m/z = 1214.2458).

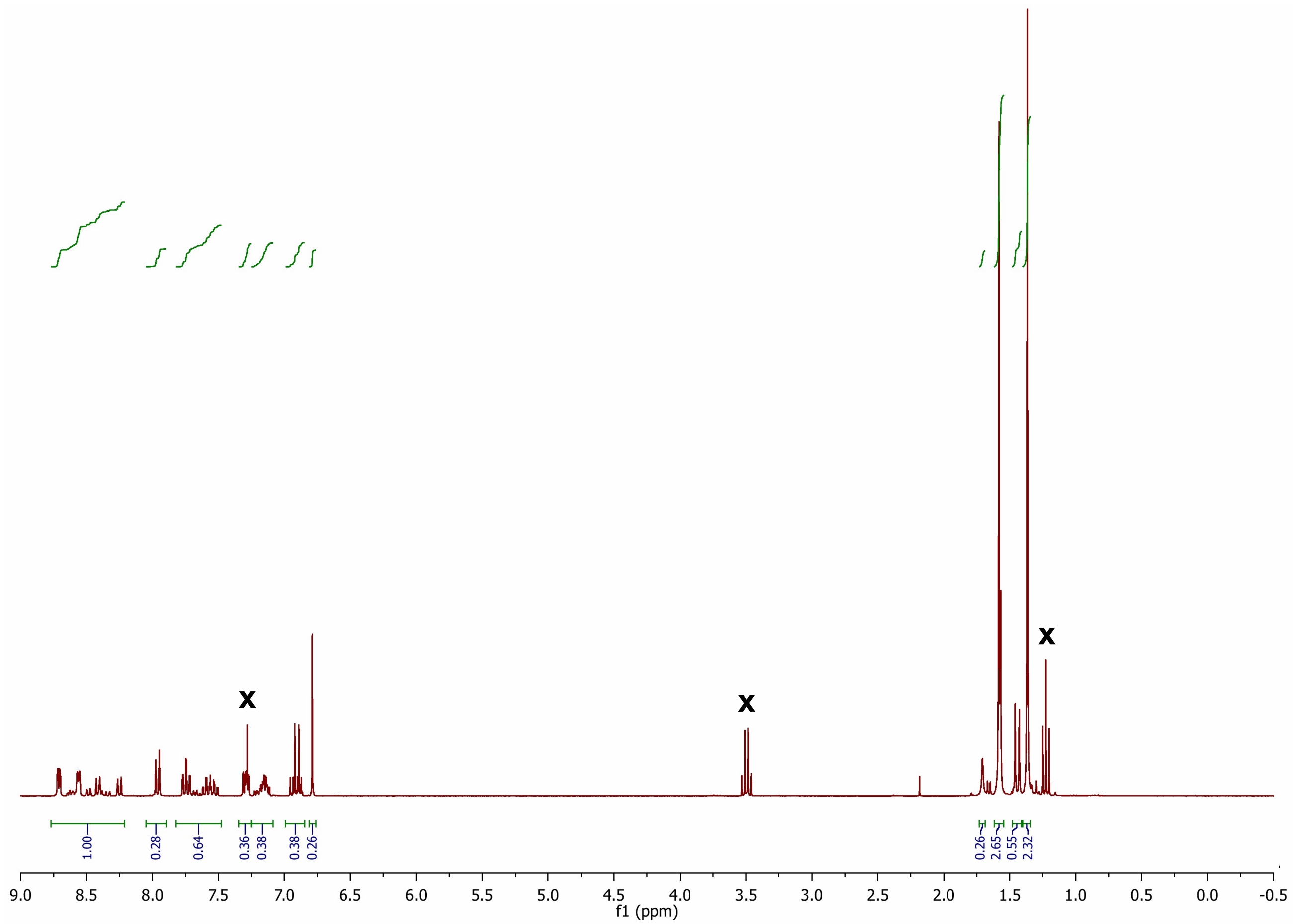

Figure S7 ^{1}H NMR spectrum of a polycrystalline sample of **1** ($CDCl_3$, 298 K). Expansions of this spectrum are shown in Figure S8.

The spectrum contains *ca* 0.3 equiv diethyl ether per "[Au*L*]" unit, which reflects that **1b** crystallizes as a diethyl ether solvate.

This spectrum was run *ca* 15 minutes after the solution was prepared. A second spectrum of the same sample recorded after 4 hours showed only minor differences from this one.

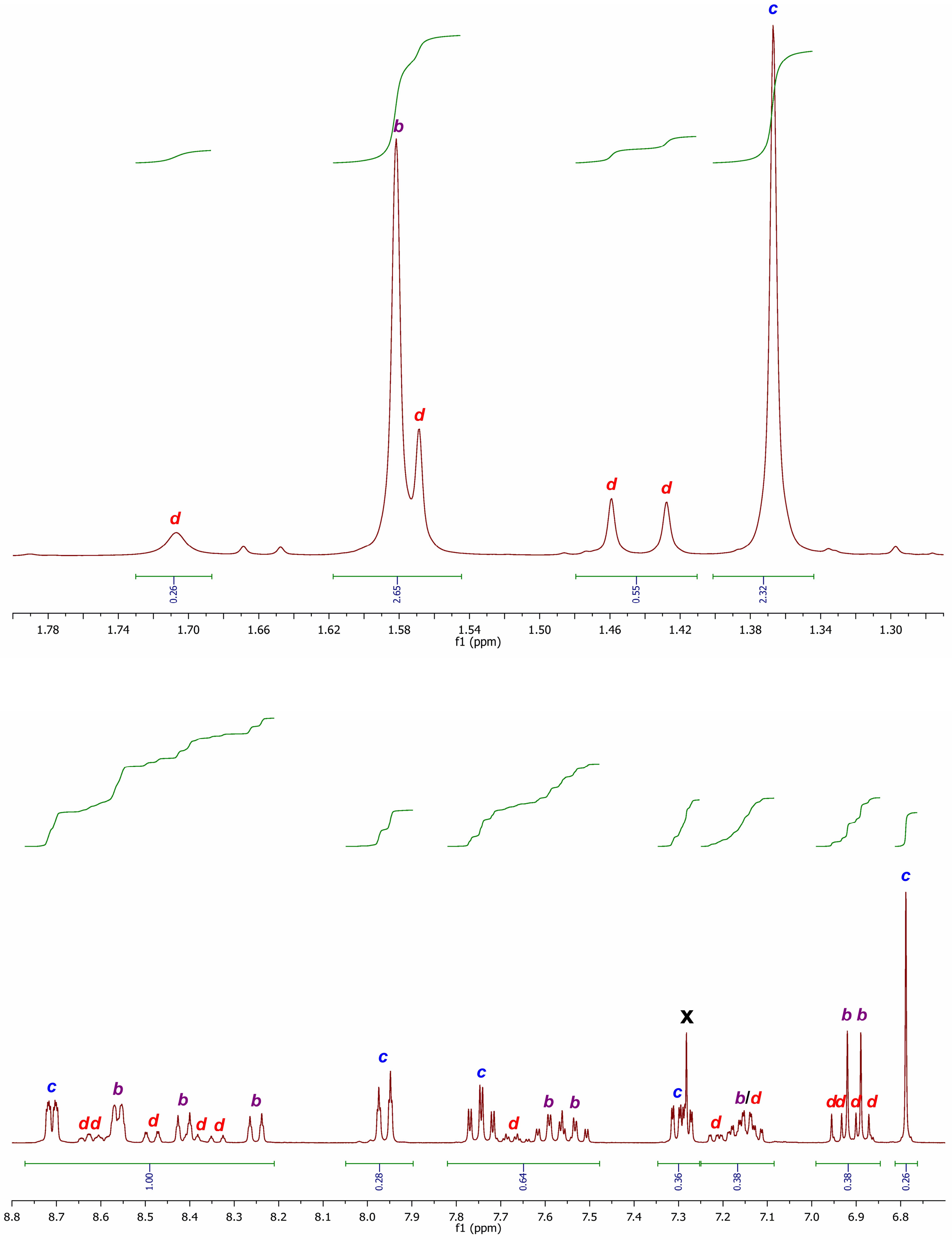

Figure S8 Expansions of the aliphatic (top) and aromatic (bottom) regions of the ^{1}H NMR spectrum of **1** (Figure S7). Peaks from the three main species present in the spectrum are labelled 'b', 'c' and 'd', as discussed on the next page.

On the basis of the peak integrals (Figure S8), the spectrum can be assigned to a mixture of three main species with two (*b*), one (*c*) and four (*d*) unique *L* environments respectively. Not all the *d* resonances are resolved in the aromatic region of the spectrum, however. The *b*:*c*:*d* ratio in the sample is approximately 1:1:0.4.

Since tetrameric $[Au_4(\mu\text{-}L)_4]$ is the main component in the mass spectrum of **1**, we assign the *b* species to the HT:HH:TH:TT isomer of that complex; *c* to the HT:HT:HT:HT form; and *d* to either the HT:HT:TH:HT or the HH:TH:TH:TT isomer (Chart S2). Isomer *b* was observed crystallographically, in **1b**·xEt$_2$O (Figure 1, main article and Figure S2).

Another species with four unique *L* environments, comprising *ca* 5 % of the sample, is also apparent in the *tert*butyl region of the spectrum (Figure S8). This is presumably the other isomer without internal symmetry, proposed for species *d* in Chart S2.

An alternative assignment of isomer *c* could be the trinuclear complex **1a** (Chart S1 and Figure S1). That seems less likely however, since **1a** was not identified in the mass spectrum of the compound (Figure S6).

HT:TH:TH:HT

Chart S2 The species proposed to be the three main components in the NMR spectrum of **1** (Figure S8; H = head {pyridyl}, T = tail {*tert*butyl}). The *b* isomer is the form found in the crystal structure of **1b**·xEt$_2$O (Figure S2).

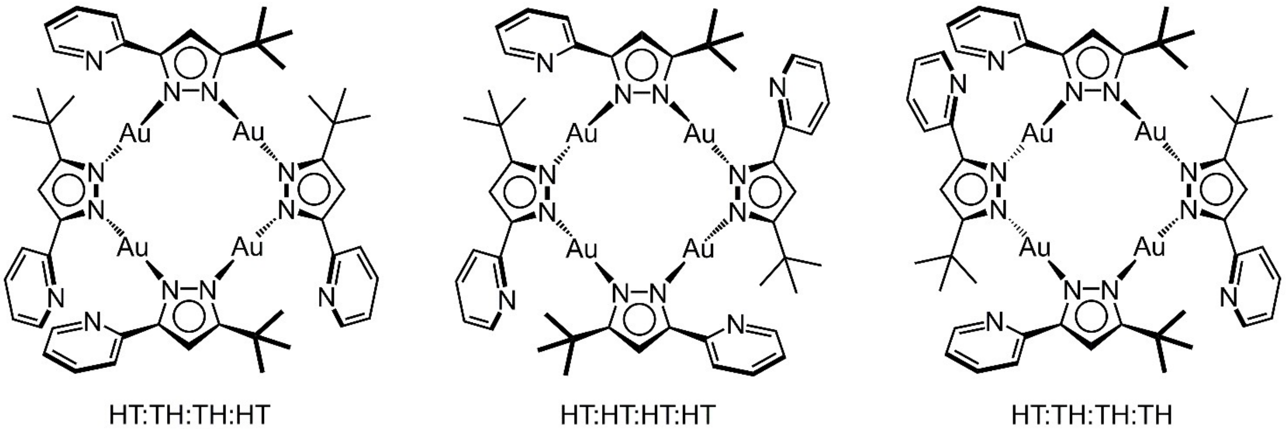

HT:TH:TH:HT **b (1b)** | HT:HT:HT:HT ***c*** | HT:TH:TH:TH ***d***

The HT sequence of substituents in each isomer is written clockwise starting from the top left corner.

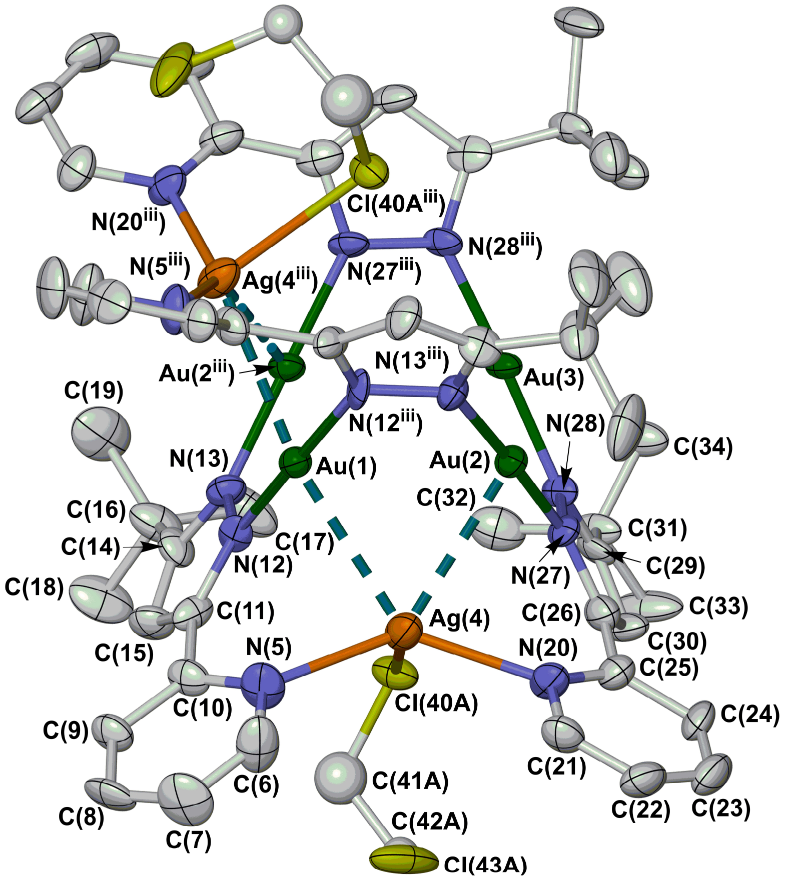

Figure S9 View of the $[Ag_2Au_4(\mu_3\text{-}L)_4(ClC_2H_4Cl)_{1.2}]^{2+}$ dication in **2**·$y$$C_2H_4Cl_2$, showing the full atom numbering scheme. Only the major disorder site for the part-occupied 1,2-dichloroethane ligands is shown. Displacement ellipsoids are at the 50 % probability level, and atoms are omitted for clarity. The view is the same as in Fig. 2 of the main article. Symmetry code: (iii) $1-x$, y, $^3/_2-z$.

Color code: C, white; Ag, orange; Au, green; Cl, yellow; N, blue.

Table S3 Selected interatomic distances and angles in **2**·$y$$C_2H_4Cl_2$ (Å, °). See Figure S9 for the atom number scheme employed. Symmetry code: (iii) $1-x$, y, $^3/_2-z$.

Au(1)–N(12)	2.006(11)	Ag(4)–Cl(40)	2.80(3)/2.87(4)/3.20(4)[a]
Au(2)–N(13^{iii})	2.010(10)	Au(1)···Au(2)	3.1645(6)
Au(2)–N(27)	2.007(9)	Au(2)···Au(3)	3.1878(7)
Au(3)–N(28)	2.011(10)	Au(1)···Ag(4)	2.9346(10)
Ag(4)–N(5)	2.252(12)	Au(2)···Ag(4)	2.9344(12)
Ag(4)–N(20)	2.276(11)		
N(12)–Au(1)–N(12^{iii})	178.1(5)	N(5)–Ag(4)–N(20)	135.8(4)
N(13^{iv})–Au(2)–N(27)	177.5(4)	N(5)–Ag(4)–Cl(40)	94.5(6)/95.9(7)/91.7(9)[a]
N(28)–Au(3)–N(28^{iii})	177.1(5)	N(20)–Ag(4)–Cl(40)	97.4(6)/101.2(7)/100.4(9)[a]

[a]This residue is disordered over three sites, with total occupancy of 0.6.

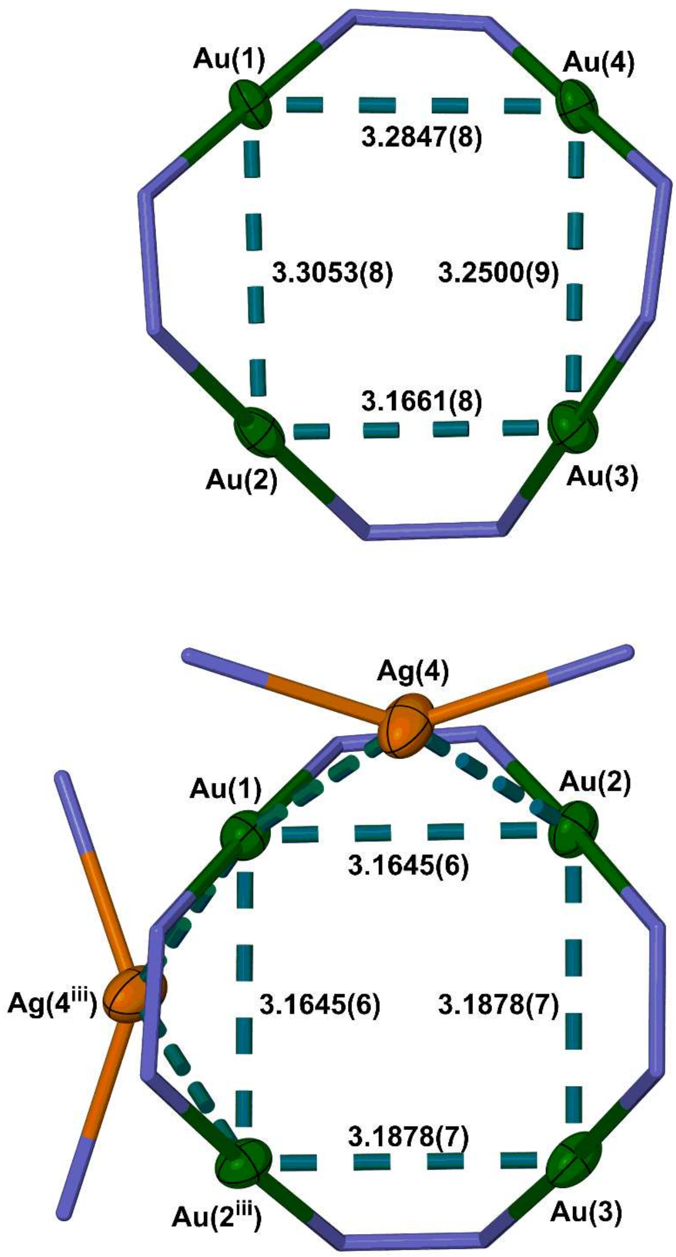

Figure S10 Comparison of the dimensions (Å) of the Au_4 cores in **1b**·$x$$Et_2O$ (top) and **2**·$y$$C_2H_4Cl_2$ (bottom). Symmetry code: (iii) x, y, $^1/_2-z$.

Color code: Ag, orange; Au, green; N, blue.

Each symmetry equivalent Ag(4) atom bridges symmetrically across an Au···Au vector, with Ag···Au distances of 2.9346(10) and 2.9344(12) Å (not shown in the Figure, for clarity).

The average Au···Au distance in **2** [3.1762(9) Å] is 0.0753(19) Å shorter than in **1b** [3.2515(17) Å], implying the aurophilic interactions in **2** may be strengthened by the metalation reaction.

There is no difference between the Au−N distances in **1b** and **2**, within experimental error (Tables S2 and S3).

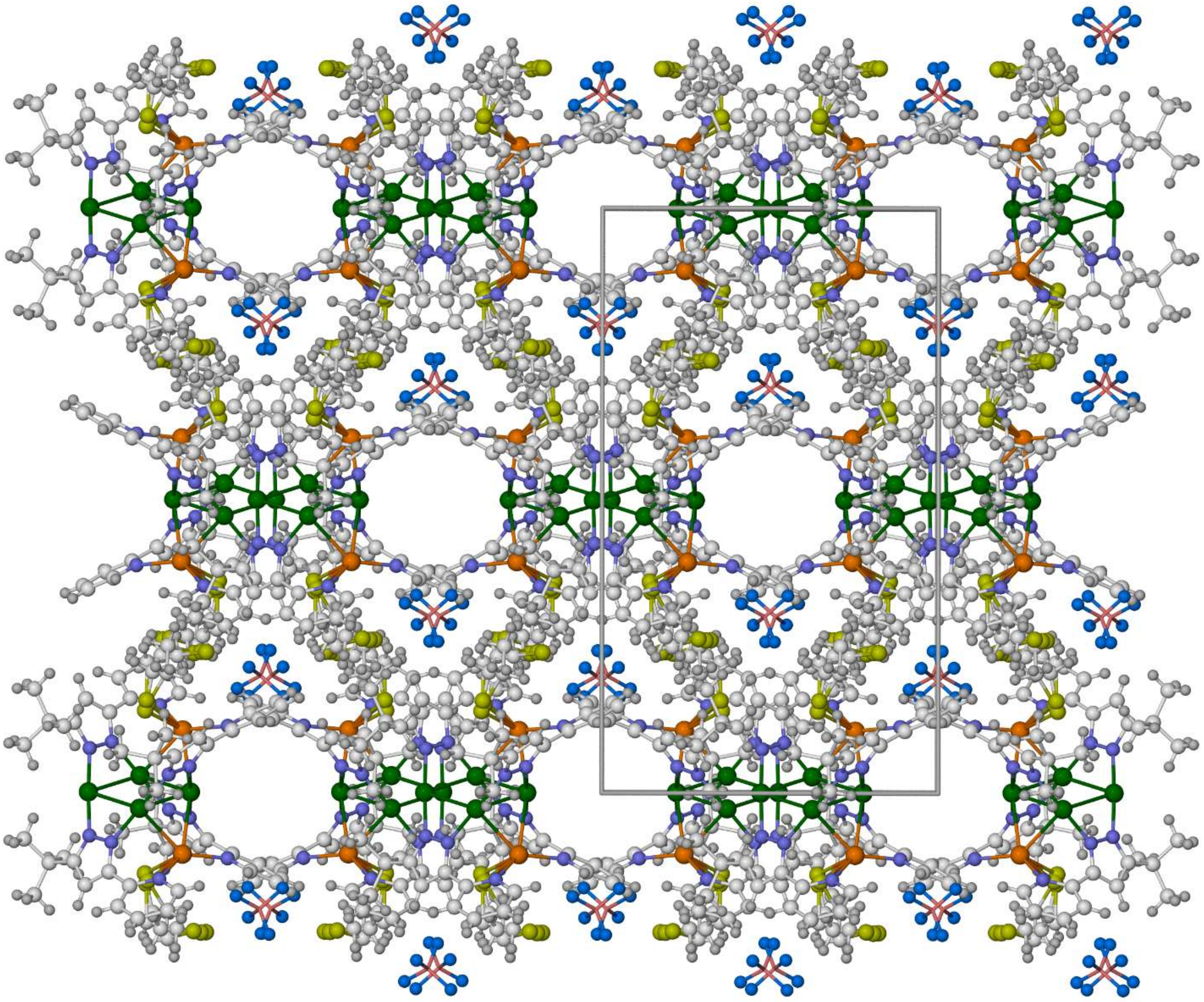

Figure S11 Packing diagram of **2**·yC$_2$H$_4$Cl$_2$, showing the channels in the lattice that are filled with disordered solvent (not shown). The view is parallel to the crystallographic [001] vector, with *b* horizontal. All atoms have arbitrary radii.

Color code: C, white; H, pale gray; Ag, orange; Au, green; B, pink; Cl, yellow; F, cyan; N, blue.

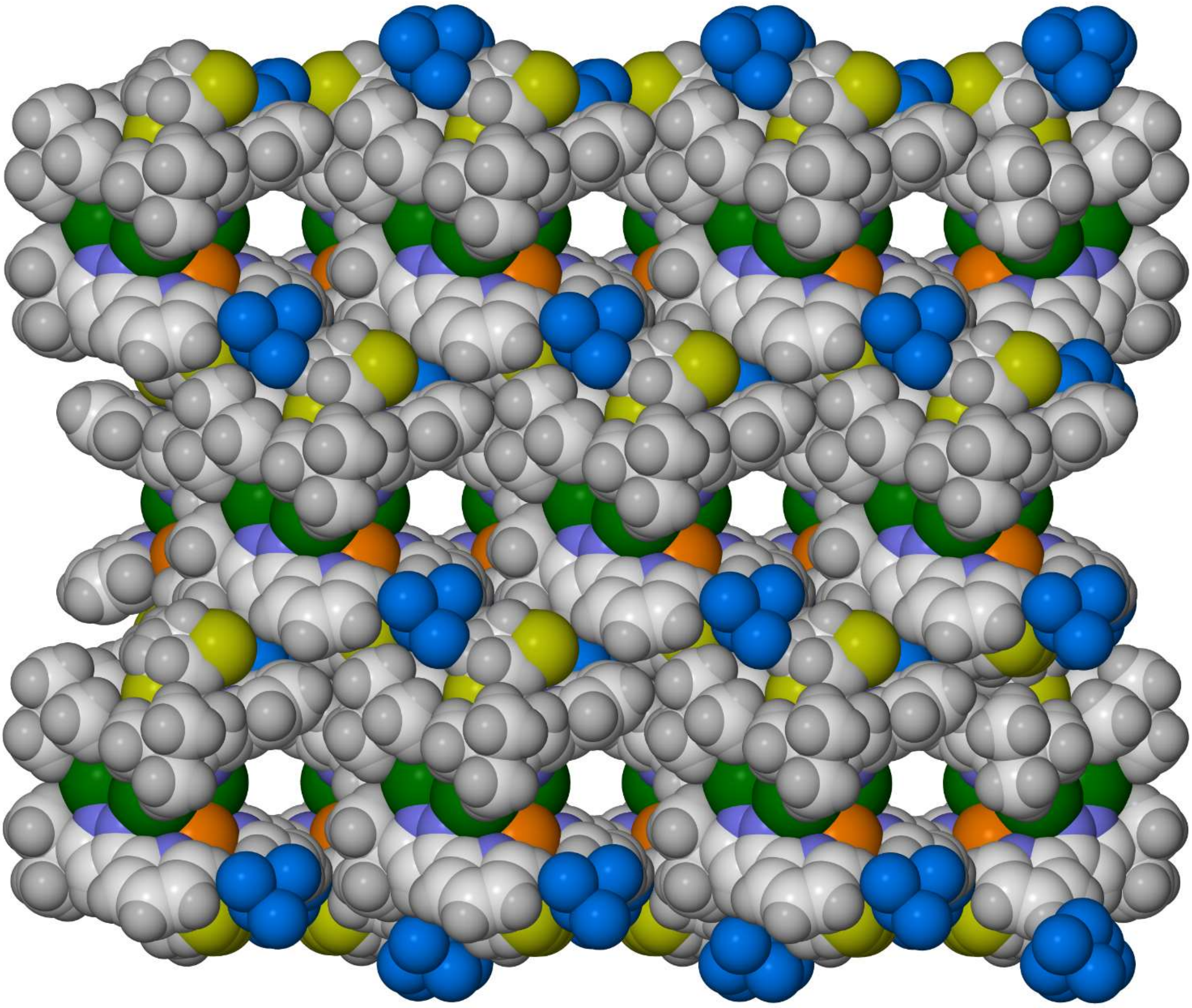

Figure S12 Space-filling packing diagram of **2**·$y$$C_2H_4Cl_2$, showing the channels in the lattice that are filled with disordered solvent (not shown). The view is the same as in Figure S11.

Color code: C, white; H, pale gray; Ag, orange; Au, green; B, pink; Cl, yellow; F, cyan; N, blue.

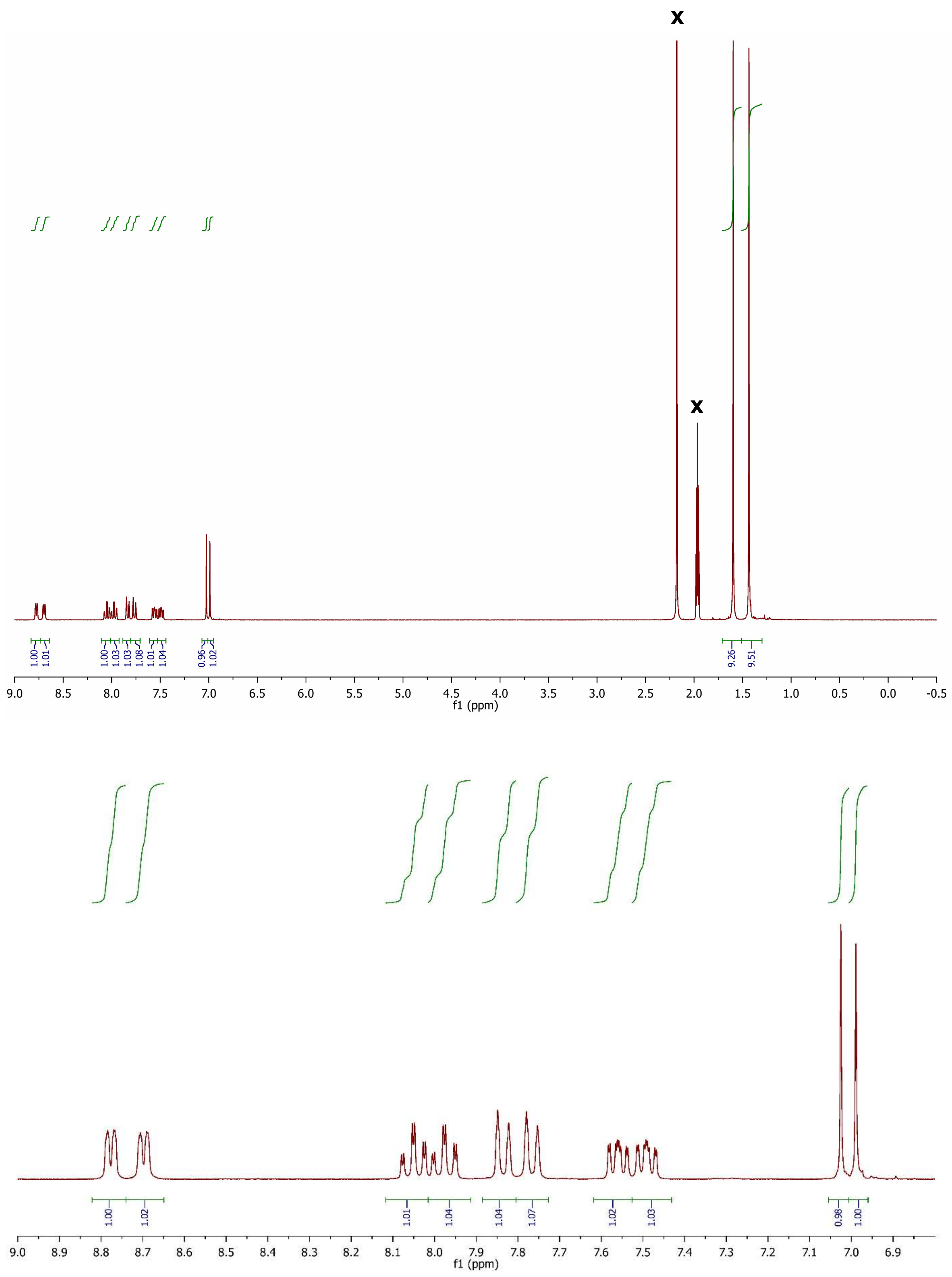

Figure S13 300 MHz ^{1}H NMR spectrum of **2** ($CDCN_3$, 298 K). Top: full spectrum. Bottom: expansion of the aromatic region.

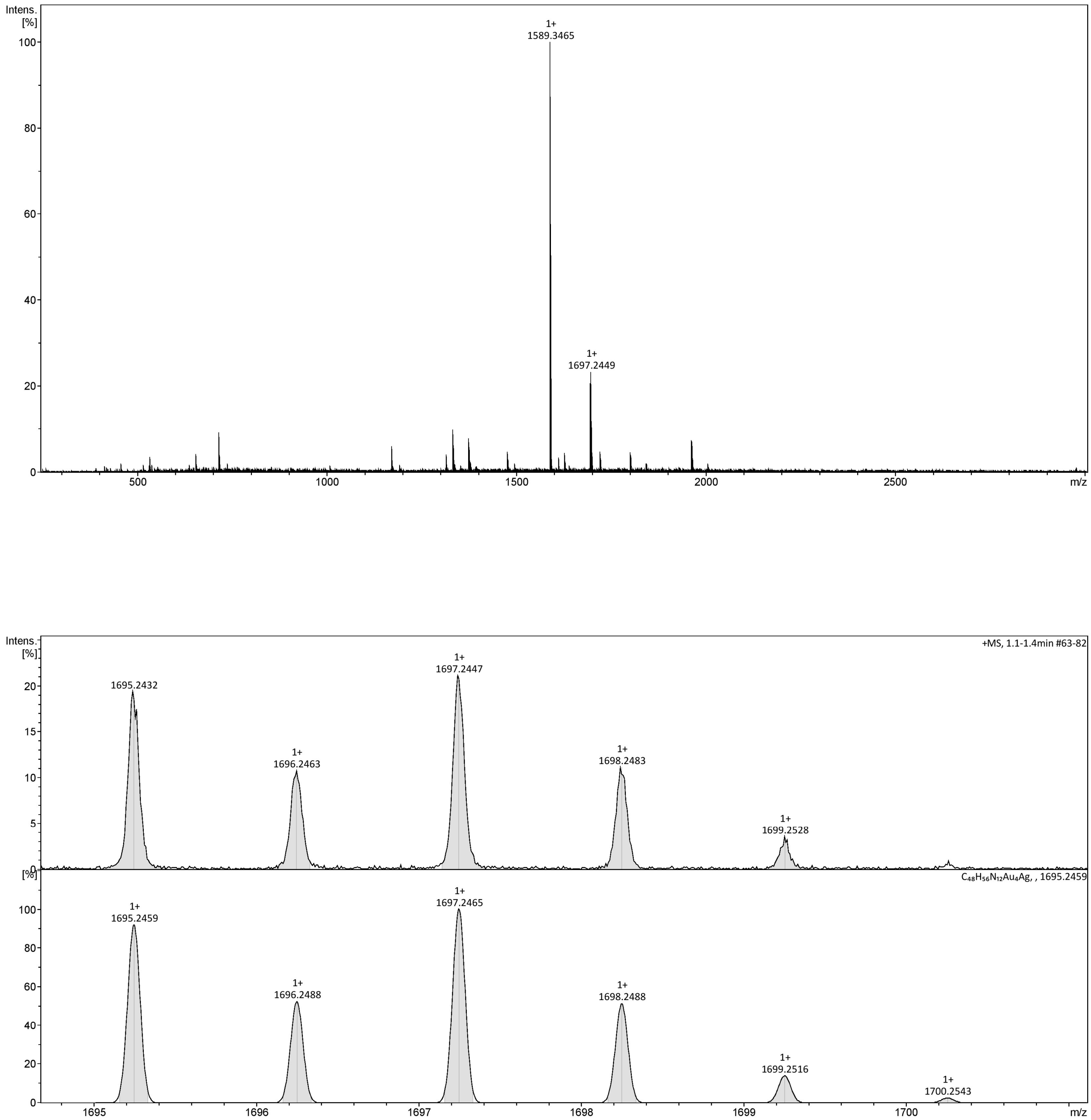

Figure S14 Top: electrospray mass spectrum of **2**. The labelled peaks correspond to the molecular ions $[HAu_4(L)_4]^+$ (m/z = 1589.3465) and $[AgAu_4(L)_4]^+$ (m/z = 1697.2449). Bottom: expansion and simulation of the $[AgAu_4(L)_4]^+$ peak.

The main fragmentation peaks in this spectrum are weaker than observed for **1** but have the same *m*/*z* values, within experimental error (Figure S6).

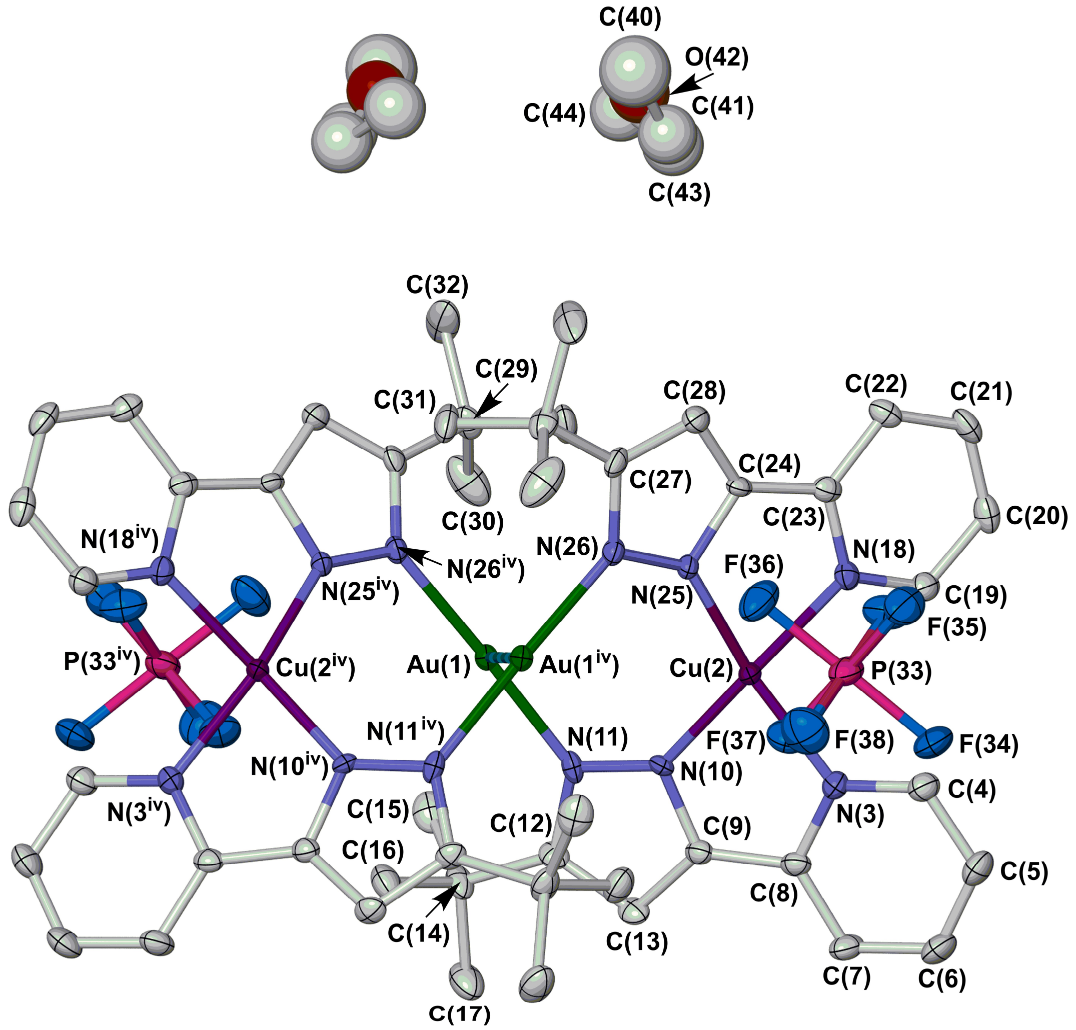

Figure S15 View of the asymmetric unit of **3**·zEt$_2$O, showing the full atom numbering scheme. The short Au···Au contact is plotted as a dashed line. Displacement ellipsoids are at the 50 % probability level, and atoms are omitted for clarity. Symmetry code: (iv) $^{1}/_{2}-x$, y, $^{1}/_{2}-z$.

Color code: C, white; Au, green; Cu, purple; F, cyan; N, blue; O, red; P, pink.

The partial diethyl ether molecules lie near a crystallographic C_2 axis, and were refined with occupancy 0.4. They lie within channels running parallel to the unit cell a axis (Figure S17).

Table S4 Selected interatomic distances and angles in **3**·zEt$_2$O (Å, °). See Figure S14 for the atom number scheme employed.

Au(1)–N(11)	1.995(5)	Cu(2)–N(18)	2.007(5)
Au(1)–N(26)	2.013(5)	Cu(2)–N(25)	1.945(4)
Cu(2)–N(3)	1.993(5)	Au(1)···Au(1^{iv})	2.9483(4)
Cu(2)–N(10)	1.965(4)	Au(1)···Cu(2)	3.8897(7)
N(11)–Au(1)–N(26)	177.97(18)	N(10)–Cu(2)–N(18)	144.95(19)
N(3)–Cu(2)–N(10)	81.83(19)	N(10)–Cu(2)–N(25)	113.06(19)
N(3)–Cu(2)–N(18)	97.1(2)	N(18)–Cu(2)–N(25)	82.29(19)
N(3)–Cu(2)–N(25)	154.58(19)		

There is also a weak secondary contact between Cu(2) and the PF_6^- ion, Cu(2)···F(37) = 3.033(4) Å. This is too long to be considered part of the inner coordination sphere of Cu(2), however.

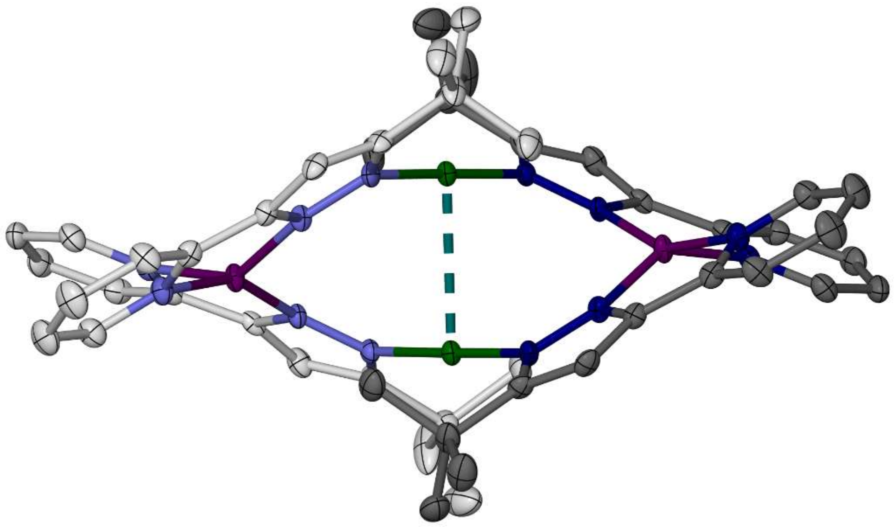

Figure S16 Alternative view of the [Cu_2Au_2(μ-L)$_4$]$^{2+}$ dication in **3**·zEt$_2$O, emphasizing its helicate conformation. The [L]$^-$ ligands from within the asymmetric unit, and the symmetry-related ligands making up the rest of the molecule, are shown with dark and pale coloration respectively. Other details as for Figure 15.

Color code: C, white or dark gray; Au, green; Cu, purple; N, pale or dark blue.

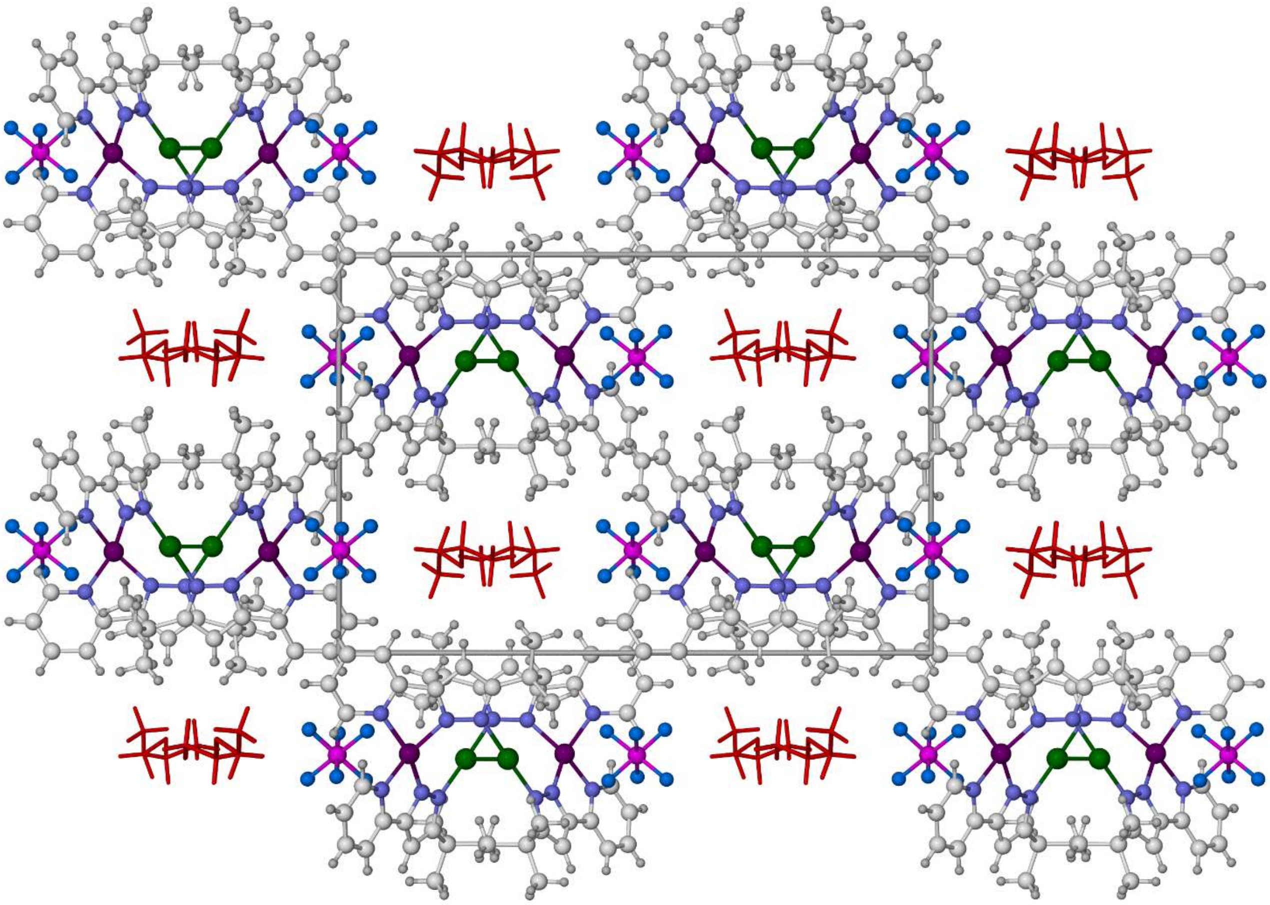

Figure S17 Packing diagram of **3**·zEt$_2$O, showing the channels containing partly-occupied diethyl molecules. The view is parallel to the [100] crystal vector with *c* horizontal, and all atoms have arbitrary radii.

Color code: C{complex}, white; Au, green; Cu, purple; F, cyan; N, blue; P, pink; diethyl ether, red.

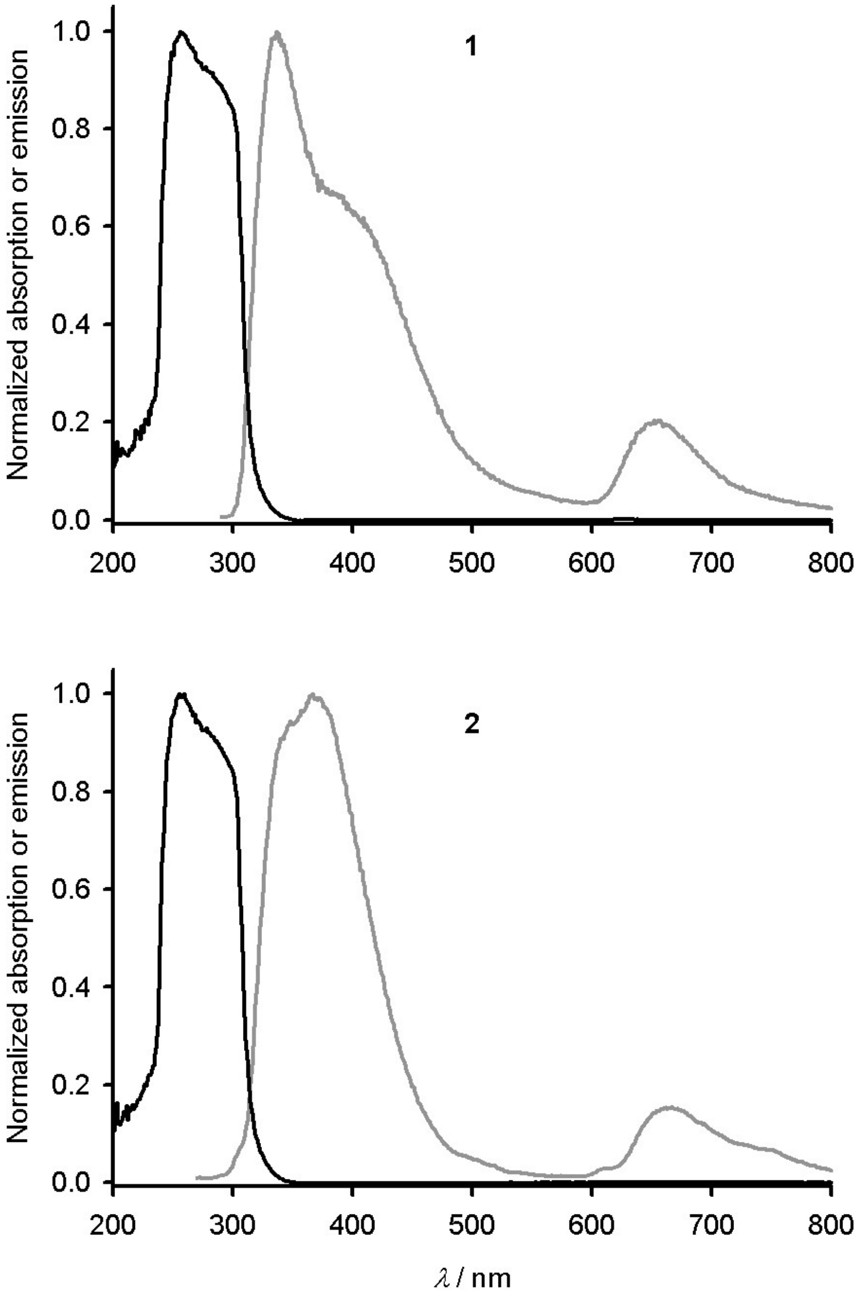

Figure S18 Full normalized absorption (black) and emission (gray) spectra for **1** (λ_{ex} = 270 nm) and **2** (λ_{ex} = 250 nm), in MeCN solution at 298 K under ambient conditions. Sample concentrations were 10^{-5} mol dm^{-3}.

Expansions of the visible region of the emission spectra are shown in Figure 4, main article.

The wavelengths of the emissions do not vary with the excitation wavelength for 250 < λ_{ex} < 320 nm, but the relative intensities of the individual emissions do depend on λ_{ex}. The strong fluorescence around 400 nm is ligand-centered,[1] while the longer wavelength emission arises from metal-to-metal charge transfer (MMCT) transitions within the coinage metal cluster core.[12]

References

(1) Yu, W.-S.; Cheng, C.-C.; Cheng, Y.-M.; Wu, P.-C.; Song, Y.-H.; Chi, Y.; Chou, P.-T. Excited-State Intramolecular Proton Transfer in Five-Membered Hydrogen-Bonding Systems: 2-Pyridyl Pyrazoles. *J. Am. Chem. Soc.* **2003**, *125*, 10800–10801.

(2) Usón, R.; Laguna, A.; Laguna, M. (Tetrahydrothiophene)Gold(I) or Gold(III) Complexes. *Inorg. Synth.* **1989**, *26*, 85–91.

(3) Usón, R.; Laguna, A.; Navarro, A.; Parish, R. V.; Moore, L. S. Synthesis and Reactivity of Perchlorate Bis(Tetrahydrothiophene)Gold(I). Gold-197 Mössbauer Spectra of Three-Coordinate Gold(I) Complexes. *Inorg. Chim. Acta* **1986**, *112*, 205–208.

(4) Kubas, G. J. Tetrakis(acetonitrile)Copper(1+) Hexafluorophosphate(1–). *Inorg. Synth.* **1990**, *28*, 68–70.

(5) See *eg*

(a) Yang, G.; Raptis, R. G. Supramolecular Assembly of Trimeric Gold(I) Pyrazolates through Aurophilic Attractions. *Inorg. Chem.* **2003**, *42*, 261–263.

(b) Omary, M. A.; Rawashdeh-Omary, M. A.; Gonser, M. W. A.; Elbjeirami, O.; Grimes, T.; Cundari, T. R.; Diyabalanage, H. V. K.; Gamage, C. S. P.; Dias, H. V. R. Metal Effect on the Supramolecular Structure, Photophysics, and Acid-Base Character of Trinuclear Pyrazolato Coinage Metal Complexes. *Inorg. Chem.*, 2005, **44**, 8200–8210.

(c) Fujisawa, K.; Ishikawa, Y.; Miyashita, Y.; Okamoto, K. Pyrazolate-Bridged Group 11 Metal(I) Complexes: Substituent Effects on the Supramolecular Structures and Physicochemical Properties. *Inorg. Chim. Acta* **2010**, *363*, 2977−2989.

(d) Earl, L. D.; Nagle, J. K.; Wolf, M. O. Tuning the Extended Structure and Electronic Properties of Gold(I) Thienyl Pyrazolates. *Inorg. Chem.* **2014**, *53*, 7106−7117.

(e) Chen, J.-H.; Liu, Y.-M.; Zhang, J.-X.; Zhu, Y.-Y.; Tang, M.-S.; Ng, S. W.; Yang, G. Halogen-Involving Weak Interactions Manifested in the Crystal Structures of Silver(I) or Gold(I) 4-Halogenated-3,5-Diphenylpyrazolato Trimers. *CrystEngComm* **2014**, *16*, 4987–4998.

(f) Woodall, C. H.; Fuertes, S.; Beavers, C. M.; Hatcher, L. E.; Parlett, A.; Shepherd, H. J; Christensen, J.; Teat, S. J.; Intissar, M.; Rodrigue-Witchel, A.; Suffren, Y.; Reber, C.; Hendon, C. H.; Tiana, D.; Walsh, A.; Raithby P. R. Tunable Trimers: Using Temperature and Pressure to Control Luminescent Emission in Gold(I) Pyrazolate-Based Trimers. *Chem. – Eur. J.* **2014**, *20*, 16933–16942.

(6) Sheldrick, G. M. *SHELXT* – Integrated Space-Group and Crystal Structure Determination. *Acta Cryst. Sect. A: Found. Adv.* **2015**, *71*, 3–8.

(7) Sheldrick, G. M. Crystal Structure Refinement with *SHELXL*. *Acta Cryst. Sect. C: Struct. Chem.* **2015**, *71*, 3–8.

(8) Barbour, L. J. X-Seed 4: Updates to a Program for Small-Molecule Supramolecular Crystallography. *J. Appl. Crystallogr.* **2020**, *53*, 1141–1146.

(9) *POVRAY* v. 3.5, Persistence of Vision Raytracer Pty. Ltd., Williamstown, Victoria, Australia, 2002.

(10) Dolomanov, O. V.; Bourhis, L. J.; Gildea, R. J.; Howard, J. A. K.; Puschmann, H. OLEX2: a Complete Structure Solution, Refinement and Analysis Program. *J. Appl. Crystallogr.* **2009**, *42*, 339–341.

(11) Spek, A. L. *PLATON SQUEEZE*: a Tool for the Calculation of the Disordered Solvent Contribution to the Calculated Structure Factors. *Acta Cryst. Sect. C.: Struct. Chem.* **2015**, *71*, 9–18.

(12) (a) Grimes, T.; Omary, M. A.; Dias, H. V. R.; Cundari, T. R. Intertrimer and Intratrimer Metallophilic and Excimeric Bonding in the Ground and Phosphorescent States of Trinuclear Coinage Metal Pyrazolates: a Computational Study. *J. Phys. Chem. A* **2006**, *110*, 5823–5830.

(b) Ni, W.-X.; Qiu, Y.-M.; Li, M.; Zheng, J.; Sun, R. W.-Y.; Zhan, S.-Z.; Ng, S. W.; Li, D. Metallophilicity-Driven Dynamic Aggregation of a Phosphorescent Gold(I)–Silver(I) Cluster Prepared by Solution-Based and Mechanochemical Approaches. *J. Am. Chem. Soc.* **2014**, *136*, 9532–9535.

(c) Watanabe, Y.; Washer, B. M.; Zeller, M.; Savikhin, S.; Slipchenko, L. V.; Wei, A. Copper(I)–Pyrazolate Complexes as Solid-State Phosphors: Deep-Blue Emission through a Remote Steric Effect. *J. Am. Chem. Soc.* **2022**, *144*, 10186–10192.
